# New Application of 1,2,4-Triazole Derivatives as Antitubercular Agents. Structure, In Vitro Screening and Docking Studies

**DOI:** 10.3390/molecules25246033

**Published:** 2020-12-19

**Authors:** Zbigniew Karczmarzyk, Marta Swatko-Ossor, Waldemar Wysocki, Monika Drozd, Grazyna Ginalska, Anna Pachuta-Stec, Monika Pitucha

**Affiliations:** 1 Faculty of Science, Siedlce University of Natural Sciences and Humanities, 08-110 Siedlce, Poland; kar@uph.edu.pl; 2 Department of Biochemistry and Biotechnology, Faculty of Pharmacy, Medical University of Lublin, 20-093 Lublin, Poland; martaswatkoossor@umlub.pl (M.S.-O.); wwysocki@uph.edu.pl (W.W.); grazyna.ginalska@umlub.pl (G.G.); 3 Independent Radiopharmacy Unit, Faculty of Pharmacy, Medical University of Lublin, 20-093 Lublin, Poland; monika.drozd@umlub.pl (M.D.); anna.pachuta@umlub.pl (A.P.-S.)

**Keywords:** 1,2,4-triazole, structure, X-ray analysis, antitubercular agent, molecular docking

## Abstract

A series of 1,2,4-triazole derivatives were synthesized and assigned as potential anti-tuberculosis substances. The molecular and crystal structures for the model compounds C1, C12, and C13 were determined using X-ray analysis. The X-ray investigation confirmed the synthesis pathway and the assumed molecular structures for analyzed 1,2,4-triazol-5-thione derivatives. The conformational preferences resulting from rotational degrees of freedom of the 1,2,4-triazole ring substituents were characterized. The lipophilicity (logP) and electronic parameters as the energy of frontier orbitals, dipole moments, NBO net charge distribution on the atoms, and electrostatic potential distribution for all structures were calculated at AM1 and DFT/B3LYP/6-311++G(d,p) level. The in vitro test was done against *M. tuberculosis* H_37_Ra, *M. phlei*, M*. smegmatis,* and *M. timereck*. The obtained results clearly confirmed the antituberculosis potential of compound C4, which turned out to be the most active against *Mycobacterium* H_37_Ra (MIC = 0.976 μg/mL), *Mycobaterium pheli* (MIC = 7.81 μg/mL) and *Mycobacerium timereck* (62.6 μg/mL). Satisfactory results were obtained with compounds C8, C11, C14 versus *Myc.* H_37_R*a*, *Myc. pheli*, *Myc. timereck* (MIC = 31.25−62.5 μg/mL). The molecular docking studies were carried out for all investigated compounds using the *Mycobacterium tuberculosis* cytochrome P450 CYP121 enzyme as molecular a target connected with antimycobacterial activity.

## 1. Introduction

Triazoles are a group of heterocyclic compounds with a five-membered ring composed of two carbon atoms and three nitrogen atoms. There are two tautomeric forms that differ in the place where hydrogen and nitrogen join—1,2,3-triazole and 1,2,4-triazole. The properties of triazoles have been used in many directions for years. Triazole derivatives, among others epoxiconazole and propiconazole are used as plant protection products [1]. Paclobutrazole and uniconazole are used as plant growth retardants [2]. Some triazole, benzotriazole, and naphthotriazole have been used as corrosion inhibitors for copper [3]. Triazole activity has also been used in medicine and biochemistry. Brassinazole is a brasynosteroid biosynthesis inhibitor [4]. Triazole derivatives have a very wide biological effect confirmed by research. They have antimicrobial activity [5], fungicidal activity [6,7], antiviral activity [8], anti-proliferative activity [9,10], cytotoxic and antitumor activity [11], antioxidant activity [12], anti-leishmanial activity [13], antitubercular activity [14], anti-inflammatory and analgesic activity [15], anticonvulsant activity [16], anti-obesitic activity [17], and antimalarial activity [18]. Studies on triazoles as inhibitors of Alzheimer’s disease have also given promising results [19]. Among the triazole derivatives are also registered substances commonly used in antifungal therapy: fluconazole, itraconazole, voriconazole, and posaconazole [20]. This disease is especially dangerous for people with reduced immunity, malnutrition, the elderly, and alcohol abusers.

A serious problem in the treatment of tuberculosis is its high infectivity, which is why the anti-tuberculosis nature of the action of triazole derivatives is an important search direction. The classic form of tuberculosis is most often caused by three species of mycobacteria: *Mycobacterium tuberculosis, Mycobacterium bovis,* and *Mycobacterium africanum*. As it turns out, the triazole derivatives have specific anti-tuberculosis activity [21,22]. Further structural modifications of these derivatives may lead to the development of anti-tuberculosis drugs.

In our paper, a series of pyridine-1,2,4-triazole derivatives were synthesized as potential anti-tuberculosis substances. All compounds were tested in vitro against *M. tuberculosis* H_37_Ra, *M. phlei*, M*. smegmatis,* and *M. timereck*. An X-ray investigation and theoretical calculations at AM1 and DFT levels were performed in order to characterize the structural, conformational, and electronic parameters of analyzed 1,2,4-triazole-5-thione derivatives. The molecular docking studies were carried out to investigate in silico the possibility of inhibition of the cytochrome P450 CYP121 enzyme associated with the mycobacterial activity.

## 2. Experimental

### 2.1. Synthesis

All chemicals used for the synthesis were purchased from TCI (Shanghai, China), Sigma-Aldrich (St. Louis, MO, USA), AlfaAesar (Haverhill, MA, USA), and POCH (Gliwice, Poland) companies and used without further purification. Melting points were determined using a Fisher-Johns block and presented without corrections. The ^1^H and ^l3^C-NMR spectra were recorded on a Bruker Avance 600 spectrometer (Bruker BioSpin GmbH, Rheinstetten, Germany) in DMSO-*d*_6_. Chemical shifts are reported in parts per million (ppm, δ scale) relative either to the internal standard (TMS) or residual solvent peak. The mass measurements were made using an Agilent Technologies liquid chromatograph 1290 coupled to an Agilent Technologies 6550 iFunnel Q-TOF LC/MS equipped with Jet Stream Technology Ion Source was employed. Flow injection analyses were performed using 0.1% formic acid in water: acetonitrile (50:50 *v/v*), mobile phase flow 0.5 mL·min^−1^. Ions were acquired in positive polarity. The mass correction was enabled, ions of *m/z* 121.0509 and 922.0098 were used as the reference ions. Instrument control, data acquisition, and analysis were performed using Agilent Mass Hunter Workstation (Santa Clara, California, United States B.07 Software. Single-charged protonated ions for all examined compounds were detected.

#### 2.1.1. General Procedure for the Synthesis of Carboxylic Acid Hydrazides (Series A)

Zero-point-zero-one mole of the corresponding carboxylic ethyl ester, 0.8 mL of 100% hydrazine hydrate, and 5 mL of anhydrous ethanol were refluxed for 2 h. On cooling, a precipitate appeared which crystallized from ethanol upon drying. Hydrazides A4–A6 were described in the previous paper [23,24,25]. Hydrazides A1–A3 were commercially available.

#### 2.1.2. General Procedure for the Synthesis of Thiosemicarbazides (Series B)

The mixture of 0.01 mol appropriate carboxylic acid hydrazide (A), 20 mL methanol, and 0.01 mol of commercially available isothiocyanates was heated for 0.5–1 h at reflux temperature. After this time the mixture was cooled and the resulting precipitate was filtered off and crystallized from ethanol. 

Compounds B1–B12 were obtained in our laboratory and described in the publication [26,27,28,29,30,31].

##### B13. 4-(2,4-Dichlorophenyl)-1-(pyridin-3-yl)acetylthiosemicarbazide

Summary formula: C_14_H_12_N_4_OSCl_2_, yield 80%, m.p. 132–133 °C. ^1^H-NMR (DMSO-*d*_6_) δ: 3.57 (s, 2H, CH_2_), 7.34–7.43 (m, 3H, CH_phenyl_), 7.69–7.71 (m, 2H CH_pyridine_), 8.44–8.51 (m, 2H, CH_pyridine_), 9.59 (s, 1H, NH), 9.86 (s, 1H, NH), 10.33 (s, 1H, NH). ^13^C-NMR (DMSO-*d*_6_) δ: 37, 123, 127, 129, 131, 132, 132, 133, 136, 137, 148, 150, 170, 182. LC-QTOF HRMS (*m/z*): calculated monoisotopic mass: 354.0109, measured monoisotopic mass: 354.0122.

##### B14. 4-(3,4-Dichlorophenyl)-1-(pyridin-4-yl)acetylothiosemicarbazide

Summary formula: C_14_H_12_N_4_OSCl_2_, yield 81%, t.t. 176–177 °C. ^1^H-NMR (DMSO-*d*_6_) δ: 3.59 (s, 2H, CH_2_), 7.33–7.37 (m, 2H, CH_pyridine_), 7.49–7.84 (m, 3H, CH_phenyl_), 8.44–8.51 (m, 2H, CH_pyridine_), 9.89 (s, 2H, 2NH), 10.31 (s, 1H, NH). ^13^C-NMR (DMSO-*d*_6_) δ: 37, 123, 126, 127, 130, 131, 137, 148, 148, 150, 151, 170, 181. LC-QTOF HRMS (*m/z*): calculated monoisotopic mass: 354.0109, measured monoisotopic mass: 354.012.

#### 2.1.3. General Procedure for the Synthesis of 1,2,4-triazole Derivatives (Series C)

In a round bottom flask, 0.01 mole of the appropriate thiosemicarbazide (B1–B14) was put and then 20–30 mL of 2% NaOH solution added. The mixture was then heated to reflux for 2 h. Then the solution was cooled and 3M HCl solution was added in small portions to precipitate the desired compound. The precipitate formed was filtered off and, after drying, purified by crystallization from methanol.

##### C1. 4-(2-Fluorphfenyl)-3-(pyridin-2-yl)-1,2,4-triazoline-5-thione

Summary formula: C_13_H_9_N_4_SF, yield 71%, m.p. 206–208 °C. ^1^H-NMR (DMSO-*d*_6_) δ: 7.26–7.56 (m, 4H, CH_phenyl_), 7.90–8.28 (m, 4H, CH_pyridine_), 14.38 (s, 1H, NH). ^13^C-NMR (DMSO-*d*_6_) δ: 116, 123, 124, 125, 131, 138, 145, 149, 156, 159, 170 [32].

##### C2. 4-(2-Chlorophenyl)-3-(pyridin-2-yl)-1,2,4-triazoline-5-thione 

Summary formula: C_13_H_9_N_4_SCl, yield: 86%, m.p. 258–260 °C. ^1^H-NMR (DMSO-*d*_6_) δ ppm: 7.37–7.41 (m, 4H, CH_phenyl_), 7.49–8.25 (m, 4H, CH_pyridine_), 14.35 (s, 1H). ^13^C-NMR (DMSO-*d*_6_) δ ppm: 123, 125, 128, 130, 130, 132, 133, 137, 145, 149, 169 [32].

##### C3. 4-(2,4-Dichlorofenylo)-3-(pyridin-2-yl)-1,2,4-triazoline-5-thione 

Summary formula: C_13_H_8_N_4_SCl_2_, yield 71%, m.p. 236–238 °C. ^1^H-NMR (DMSO-*d*_6_) δ: 7.44–7.65 (m, 4H, CH_phenyl_), 7.89–8.36 (m, 4H, CH_pyridine_), 14.49 (s, 1H, NH) [32]. 

##### C4. 4-(3,4-Dichlorofenylo)-3-(pyridin-2-yl)-1,2,4-triazoline-5-thione 

Summary formula: C_13_H_8_N_4_SCl_2_, yield 72%, m.p. 231–233 °C. ^1^H-NMR (DMSO-*d*_6_) δ: 7.44–7.89 (m, 4H, CH_phenyl_), 8.05–8.36 (m, 4H, CH_pyridine_), 14.49 (s, 1H, NH) [32]. 

##### C5. 4-(2-Chlorofenylo)-3-(pyridin-3-yl)-1,2,4-triazoline-5-thione 

Summary formula: C_13_H_9_N_4_SCl, yiled 76%, m.p. 250–252 °C. ^1^H-NMR (DMSO-*d*_6_) δ: 7.39–7.79 (m, 4H, CH_phenyl_), 8.52–8.63 (m, 4H, CH_pyridine_), 14.40 (s, 1H, NH). ^13^C-NMR (DMSO-*d*_6_) δ: 122, 124, 129, 130, 132, 135, 148, 151, 169 [32]. 

##### C6. 4-(4-Nitrofenylo)-3-(pyridin-3-yl)-1,2,4-triazoline-5-thione 

Summary formula: C_13_H_9_N_5_O_2_S, yield 63%, m.p. 219–220 °C. ^1^H-NMR (DMSO-*d*_6_) δ: 7.33–7.42 (m, 4H, CH_phenyl_), 8.64–8.67 (m, 4H, CH_pyridine_), 14.41 (s, 1H, NH). ^13^C-NMR (DMSO-*d*_6_) δ: 124, 125, 130, 138, 141, 145, 147, 149, 164 [32].

##### C7. 4-(2,4-Dichlorofenylo)-3-(pyridin-3-yl)-1,2,4-triazoline-5-thione

Summary formula: C_13_H_8_N_4_SCl_2_, yiled 78%, m.p. 248–249 °C. ^1^H-NMR (DMSO-*d*_6_) δ: 7.43–7.90 (m, 4H, CH_phenyl_), 8.56–8.65 (m, 4H, CH_pyridine_), 14.44 (s, 1H, NH). ^13^C-NMR (DMSO-*d*_6_) δ: 122, 124, 129,130, 131, 133, 135, 136,148, 151, 169 [32]. 

##### C8. 4-(3,4-Dichlorofenylo)-3-(pyridin-3-yl)-1,2,4-triazoline-5-htione 

Summary formula: C_13_H_8_N_4_SCl_2_, yield 81%, m.p. 184–185 °C. ^1^H-NMR (DMSO-*d*_6_) δ: 7.49–8.09 (m, 4H, CH_phenyl_), 8.49–8.87 (m, 4H, CH_pyridine_), 14.53 (s, 1H, NH). ^13^C-NMR (DMSO-*d*_6_) δ: 124, 125, 126, 128, 129, 130, 131, 137, 138, 139, 143, 144, 146, 147, 148 [32].

##### C9. 4-(2-Fluorofenylo)-3-(pyridin-4-yl)-1,2,4-triazoline-5-thione

Summary formula: C_13_H_9_N_4_S, yiled 88%, m.p. 222–224 °C. ^1^H-NMR (DMSO-*d*_6_) δ: 7.28–7.77 (m, 4H, CH_phenyl_), 8.60–8.69 (m, 4H, CH_pyridine_), 14.62 (s, 1H, NH). ^13^C-NMR (DMSO-*d*_6_) δ: 117, 121, 126, 131, 133, 148, 150, 155, 158, 170 [32]. 

##### C10. 4-(2,4-Dichlorophenyl)-3-(pyridin-4-yl)-1,2,4-triazoline-5-thione 

Summary formula: C_13_H_8_N_4_SCl_,_ yield 79%, m.p. 264–266 °C. ^1^H-NMR (DMSO-*d*_6_) δ: 7.30–7.99 (m, 3H, CH_phenyl_), 8.69–8.72 (m, 4H, CH_pyridine_), 14.64 (s, 1H, NH). ^13^C-NMR (DMSO-*d*_6_) δ: 121, 129, 130, 131, 132, 134, 136, 148, 150, 169 [32]. 

##### C11. 4-Phenyl-3-(pyridin-2-yl)-1,2,4-triazoline-5-thione 

Summary formula: C_13_H_10_N_4_S, yield 88%, m.p. 220–221 °C. ^1^H-NMR (DMSO-*d*_6_) δ ppm: 7.24–7.37 (m, 2H, CH_pyridine_), 7.38–7.42 (m, 5H, CH_phenyl_), 7.79–7.88 (m, 2H, CH_pyridine_), 8.30–8.35 (m, 2H, CH_pyridine_), 14.25 (s, 1H, NH) [33]. 

##### C12. 4-(4-Methoxyphenyl)-3-(pyridin-3-yl)-1,2,4-triazoline-5-thione 

Summary formula: C_14_H_12_N_4_OS, yield 79%, m.p. 248–250 °C. ^1^H-NMR (DMSO-*d*_6_) δ: 2.53 (s, 3H, CH_3_), 7.05–7.75 (m, 4H, CH_phenyl_), 8.51–8.62 (m, 4H, CH_pyridine_), 14.20 (s, 1H, NH) [31]. 

##### C13. 4-(2,4-Dichlorophenyl)-3-(pyridin-3-ylmethyl)-1,2,4-triazoline-5-thione

Summary formula: C_14_H_10_N_4_SCl_2_, yield 68%, m.p. 208–210 °C. ^1^H-NMR (DMSO-*d*_6_) δ: 3.35 (s, 2H, CH_2_), 7.23–7.64 (m, 4H, CH_phenyl_), 7.81–8.42 (m, 4H, CH_pyridine_), 13.95 (s, 1H, NH). LC-QTOF HRMS (*m/z*): calculated monoisotopic mass: 336.0003, measured monoisotopic mass: 336.0017.

##### C14. 4-(3,4-Dichlorophenyl)-3-(pyridin-4-ylmethyl)-1,2,4-triazoline-5-thione

Summary formula: C_14_H_10_N_4_SCl_2_, yield 72%, m.p. 310–312 °C. ^1^H-NMR (DMSO-*d*_6_) δ: 4.14 (s, 2H, CH_2_), 7.49–7.99 (m, 4H, CH_phenyl_), 8.30–8.83 (m, 4H, CH_pyridine_), 13.97 (s, 1H, NH). LC-QTOF HRMS (*m/z*): calculated monoisotopic mass: 336.0003, measured monoisotopic mass: 336.0006.

### 2.2. X-ray Structure Determination

X-ray data of C1, C12, and C13 were collected on the KUMA Diffraction KM-4 CCD diffractometer; MoKα (λ = 0.71073 Å) radiation, ω scans, T = 293(2) K; crystal sizes 0.20 × 0.15 × 0.10 mm for C1, 0.30 × 0.10 × 0.10 mm for C12 and 0.40 × 0.40 × 0.10 mm for C13, absorption correction: multi-scan CrysAlisPro [34], Tmin/Tmax of 0.7963/1.0000 for C1, 0.9166/1.0000 for C12 and 0.8839/1.0000 for C13. The structures were solved by direct methods using SHELXS97 [35] and refined by full-matrix least-squares with SHELXL-2014/7 [35]. The N-bound H atoms were located by difference Fourier synthesis and refined freely. The remaining H atoms were positioned geometrically and treated as riding on their parent C atoms with C-H distances of 0.93 Å (aromatic) and 0.97 Å (CH_2_). All H atoms were refined with isotropic displacement parameters taken as 1.5 times those of the respective parent atoms. All calculations were performed using the WINGX version 1.64.05 package [36]. CCDC-2044947 for C1, CCDC-2044948 for C12, and CCDC-2044949 for C13 contain the supplementary crystallographic data for this paper. These data can be obtained free of charge at www.ccdc.cam.ac.uk/conts/retrieving.html [or from the Cambridge Crystallographic Data Centre (CCDC), 12 Union Road, Cambridge CB2 1EZ, UK; fax: +44(0)-1223-336-033; email: deposit@ccdc.cam.ac.uk].

Crystal data of **C1**: C_13_H_9_N_4_SF, *M* = 272.30, triclinic, space group *P*$\bar{1}$, *a* = 7.0908(6), *b* = 7.8326(7), *c* = 12.4147(10) Å, α = 71.722(8), β = 74.428(7), γ = 87.846(7)°, *V* = 629.87(10) Å^3^, *Z* = 2, *d*_calc_ = 1.436 Mg·m^−3^, *F*(000) = 280, *μ*(MoK*α*) = 0.259 mm^−1^, *T* = 293K, 4337 measured reflections (*θ* range 1.79–29.11°), 2845 unique reflections, final *R* = 0.056, *wR* = 0.142, *S* = 1.080 for 2092 reflections with *I* > 2*σ*(*I*). 

Crystal data of **C12**: C_14_H_12_N_4_OS, *M* = 284.34, monoclinic, space group *P*2_1_*/c*, *a* = 9.3878(19), *b* = 9.858(2), *c* = 14.600(3) Å, β = 90.59(3)°, *V* = 1351.1(5) Å^3^, *Z* = 4, *d*_calc_ = 1.398 Mg m^−3^, *F*(000) = 592, *μ*(Mo K*α*) = 0.240 mm^−1^, *T* = 293K, 5862 measured reflections (*θ* range 2.17–29.23°), 3073 unique reflections, final *R* = 0.042, *wR* = 0.096, *S* = 1.113 for 2286 reflections with *I* > 2*σ*(*I*).

Crystal data of **C13**: C_14_H_10_N_4_SCl_2_, *M* = 337.22, triclinic, space group *P*$\bar{1}$, *a* = 8.4234(10), *b* = 8.8584(9), *c* = 11.251(2) Å, α = 81.356(13), β = 83.534(12), γ = 66.032(11)°, *V* = 757.21(19) Å^3^, *Z* = 2, *d*_calc_ = 1.479 Mg m^−3^, *F*(000) = 344, *μ*(MoK*α*) = 0.564 mm^−1^, *T* = 293K, 4996 measured reflections (*θ* range 1.83–28.93°), 4996 unique reflections, final *R* = 0.040, *wR* = 0.102, *S* = 0.903 for 3212 reflections with *I* > 2*σ*(*I*).

### 2.3. Theoretical Calculations

The energy, geometry, and electronic parameters (frontier orbitals, dipole moments, NBO net charge distribution on the atoms, and electrostatic potential distribution) for all structures **C1**–**C14** were calculated with GAUSSIAN 03 [37] at the DFT/B3LYP level with 6–311++G(d,p) basis set. The structures were fully optimized and the initial geometries were built from the crystallographic data of **C1**, **C12,** and **C13**. The visualization of theoretical calculation results was made using GaussView [38]. The partition coefficients, logP, calculated by summing up all individual atom contributions to the lipophilicity of molecule [39] were obtained from the QSAR properties procedure implemented in HyperChem Professional package ver. 8.0.10 [40]. The conformational analysis for **C3**, **C7,** and **C10** was performed using the semiempirical AM1 method implemented in GAUSSIAN 03.

### 2.4. Antimycobacterial Assay

Four strains of mycobacteria, *M. tuberculosis* H_37_Ra, *M. phlei, M. smegmatis,* and *M. timereck*, were used for the study. The strains were donated by National Tuberculosis and Lung Diseases Research Institute (Warsaw, Poland). The microorganisms were stored on Lewenstein–Jensen media at 4 °C, then transferred to vials containing Middlebrook 7H9 broth supplemented with sodium chloride, bovine albumin, dextrose, and catalase (ADC-BBL/Becton-Dickinson, USA) and grown at 37 °C. The MIC values (minimal inhibitory concentration) for the tested compounds were determined by the log2 dilution method [41]. (Turnidge and Jorgensen, 1999). The MIC value was estimated visually as a lack of turbidity in the tube, indicating inhibition of microbial growth. The stock solutions of the tested compounds were freshly prepared in dimethyl sulfoxide (DMSO). The concentrations of test substances were in the range of 500–1.95 μg/mL. All procedure was described earlier [29].

### 2.5. Molecular Docking

The crystal structure of *Mycobacterium tuberculosis* cytochrome P450 CYP121 in complex with 1-((4-chlorophenyl)methyl)-4-(3-imidazol-1-ylpropyl)piperazin-2-one (9KE) and molecule of heme as a prosthetic group obtained from Protein Data Bank (PDB ID: 5O4K) [42] was used in docking studies for C1–C14 performed by the GOLD Suite v.5.7.3 software [43]. Preparation of enzyme for docking procedure including the addition of hydrogens, removal of water molecules, extraction of original ligand 9KE from the protein active site were done with GOLD as per default settings. The binding site of the ligand 9KE in cytochrome P450 CYP121 was used as an active site for docked molecules after removing 9KE from it. The selection of atoms in the active site within 6 Å of the original ligand was chosen. In docking stimulations, the ligand was kept flexible but the amino acid residues of the enzyme were held rigid. The initial molecular structures of C1–C14 were obtained from DFT calculation. For the simulation runs default parameter values were taken. ChemPLP as an empirical fitness function optimized for pose prediction was selected as the scoring function [43,44]. The scoring function is used to quantify binding affinity based on the ligand binding pose. The re-docking of 9 KE into the binding site of cytochrome P450 CYP121 as reference docking gave the RMSD value of 0.907 Å showing that the binding mode was successful. Analysis of the docking results was carried out using Hermes v.1.10.3 [43].

## 3. Results and Discussion

### 3.1. Chemistry

**Scheme 1:**
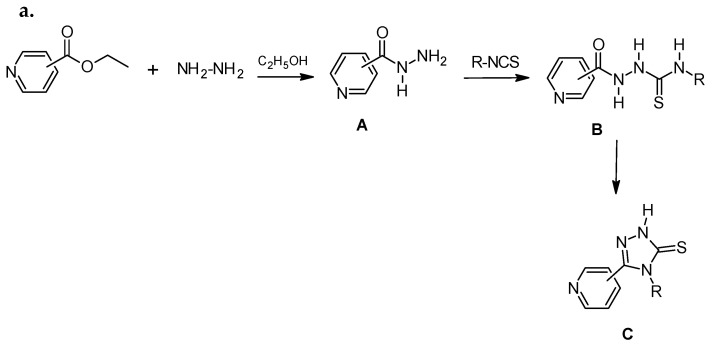

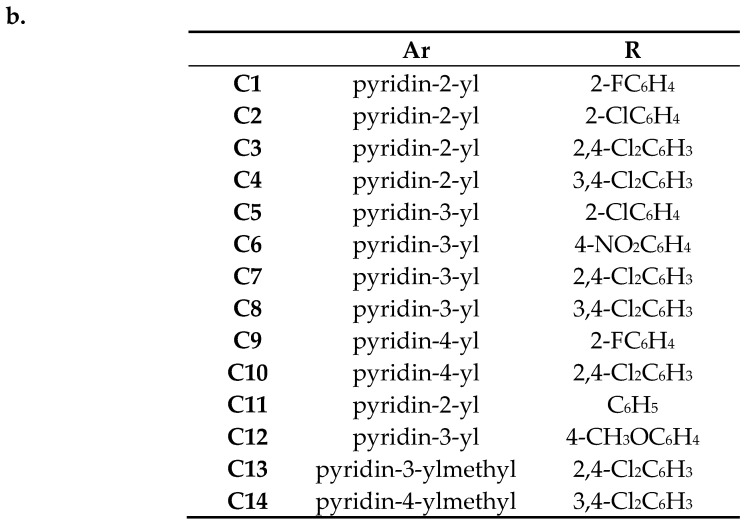
Synthesis of 1,2,4-triazole derivatives (series C) (**a**) and list of substituents (**b**).

Hydrazides of carboxylic acids were used for planned synthesis. Among them were: 2-pyridinecarboxylic acid hydrazide (A1), 3-pyridinecarboxylic acid hydrazide (A2), 4-pyridinecarboxylic acid hydrazide (A3), 2-pyridneacetic acid (A4), 3-pyridineacetic acid (A5), and 4-pyridineacetic acid hydrazide (A6). Hydrazide (A1–A3) is available commercially but hydrazide (A4–A6) was obtained by the method described in the literature [23,24,25]. The title compounds were obtained in the reaction cyclization of appropriate thiosemicarbazide derivatives (B1–B14) in an alkaline medium. Compounds B1–B10 were obtained in our laboratory by the reaction of the corresponding hydrazide (A1–A6) with commercially available isothiocyanates: 2-fluorophenyl, 2-chlorophenyl, 2,4-dichlorophenyl, 3,4-dichlorophenyl, 4-nitrophenyl, 4-methoxyphenyl, and phenyl isothiocyanate. The reaction was carried out at the boiling point of methanol during 0.5–1 h. Next, the obtained derivatives were cyclized in an alkaline medium to a 1,2,4-triazole system (C1–C14) (Scheme 1).

**Figure 1:**
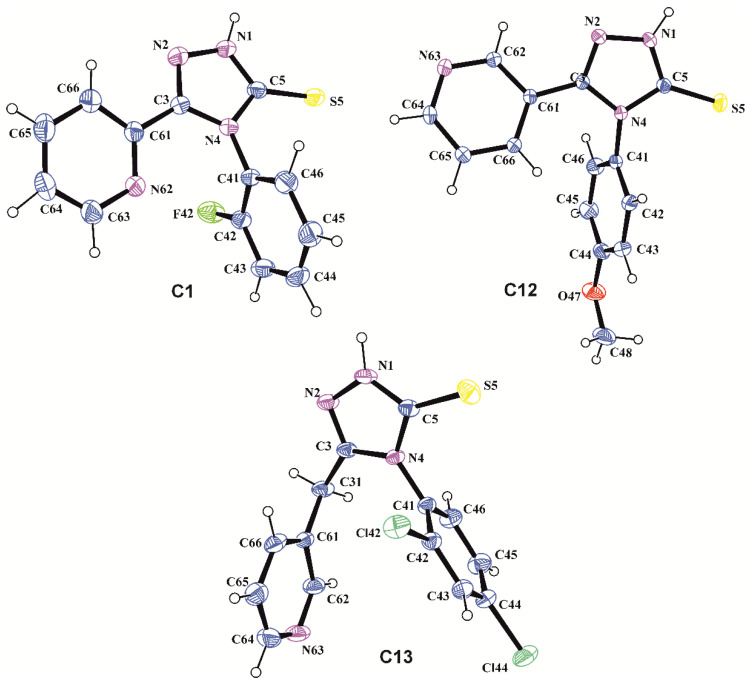
The molecular structures of C1, C12, and C13 with atom labeling and displacement ellipsoids drawing at the 30% probability level.

The structure of the obtained compounds was confirmed by spectral analysis. For the new compounds mass spectrometry was made (Table S1) and for compounds **C1**, **C12**, **C13**—the crystallography analysis (Figure 1 and Figure S1). Supplementary Materials contains additional information.

### 3.2. X-ray Analysis 

The molecular and crystal structures for C1, C12, and C13 were determined using the X-ray analysis method to confirm the assumed molecular structures, identify their tautomeric form, and find conformational preferences and intermolecular interactions observed in the crystalline state. The crystal and molecular structures of 4-(2-chlorophenyl)-3-(pyridin-2-yl)-1,2,4-triazolin-5-thione, C2, and 4-(2,4-dichlorofenylo)-3-(pirydyn-2-yl)-1,2,4-triazolino-5-tion, C3, were reported earlier [26]. The structure and conformation of the molecules C1, C12, and C13 in the crystal are presented in Figure 1.

The bond length C5–S5 of 1.669(5), 1.663(3), and 1.665(3) Å for C1, C12, and C13, respectively, typical for the thione group (1.671(24) Å [45]) and the position of the H atom in the immediate vicinity of N1 atom of 1,2,4-triazole ring in the difference electron-density map indicate unequivocally that the molecule C1, C12, and C13, similar to C2 and C3 [26], exist in N1-amino/S5-thione tautomeric form in the crystalline state. The 1,2,4-triazole, pyridine, and benzene rings in all investigated molecules are planar to within 0.007(3), 0.012(4) and 0.009(3) Å in C1, 0.0011(19), 0.008(2), and 0.008(2) Å in C12 and 0.010(2), 0.004(3) and 0.006(3) Å in C13, respectively. Approximately perpendicular position of the phenyl substituent in relation to the 1,2,4-triazole ring described by the torsion angle C3–N4–C41–C42 of 88.8(3)° in C1, −107.2(3)° in C12 and −107.4(2)° in C13 is forced by the steric effect of 2-F, 4-methoxy and 2,4-Cl substituents in the benzene ring in C1, C12, and C13, respectively. The methoxy group in C12 is located almost coplanar with the plane of the benzene ring, which shows the torsion angle C43–C44–O47–C48 of 3.4(4)°. The pyridin-2-yl substituent in C1 adopts the cis conformation relative to N2–C3 bond in the 1,2,4-triazole ring, while in the pyridin-3-yl group in C12 the trans conformation is observed. This is confirmed by the value of the torsion angle N4–C3–C61–N(C)62 equal to −16.7(4)° in C1 and −157.5(2)° in C12. The pyridin-3-ylmethyl substituent in C13 adopts the *gauche-gauche* conformation with respect to the 1,2,4-triazole ring as shown by the torsion angles N4–C3–C31–C61 and C3–C31–C61–C62 of 65.9(4) and −138.6(3)°, respectively.

The tautomeric form observed in the crystalline state is stabilized by the intermolecular hydrogen bonds: N1–H1…S5 in C1 and N1–H1…N63 in C12 and C13 (Table 1). 

In the crystal structure of C1 the inversion-related molecules are linked into molecular dimers through intermolecular hydrogen bond N1–H1…S5. In the crystal of C12, the molecules related by c glide planes connect in molecular chains parallel to Z crystallographic axis via the intermolecular hydrogen bond N1–H1…N63. Moreover, the 1,2,4-triazole rings belonging to the inversion related molecules partially overlap each other with the π…π distance of 3.3651(8) Å characteristic for the overlapping π-aromatic ring systems. In the crystal structure of C13 the molecules related by translations *a* and *b* form molecular chains parallel to [110] crystallographic direction by N1–H1…N63 hydrogen bond. Moreover, in this crystal, the net of dimer chains formed by the weak intermolecular hydrogen bonds C–H…X (X = S, N) is observed (Table 1). The structural motifs formed by molecules through intermolecular hydrogen bonds in crystals of C1, C11, and C13 are presented in Figure S1 (Supplementary Materials).

It should be noted that the intermolecular interactions observed in the crystal may reflect the interactions of the molecules of the tested compounds with the molecular target related to their biological activity.

### 3.3. Theoretical Calculations

The conformation of the molecules is an important factor associated with biological activity [46] and this is one of the key factors in the interaction of a molecule with a molecular target.

**Figure 2:**
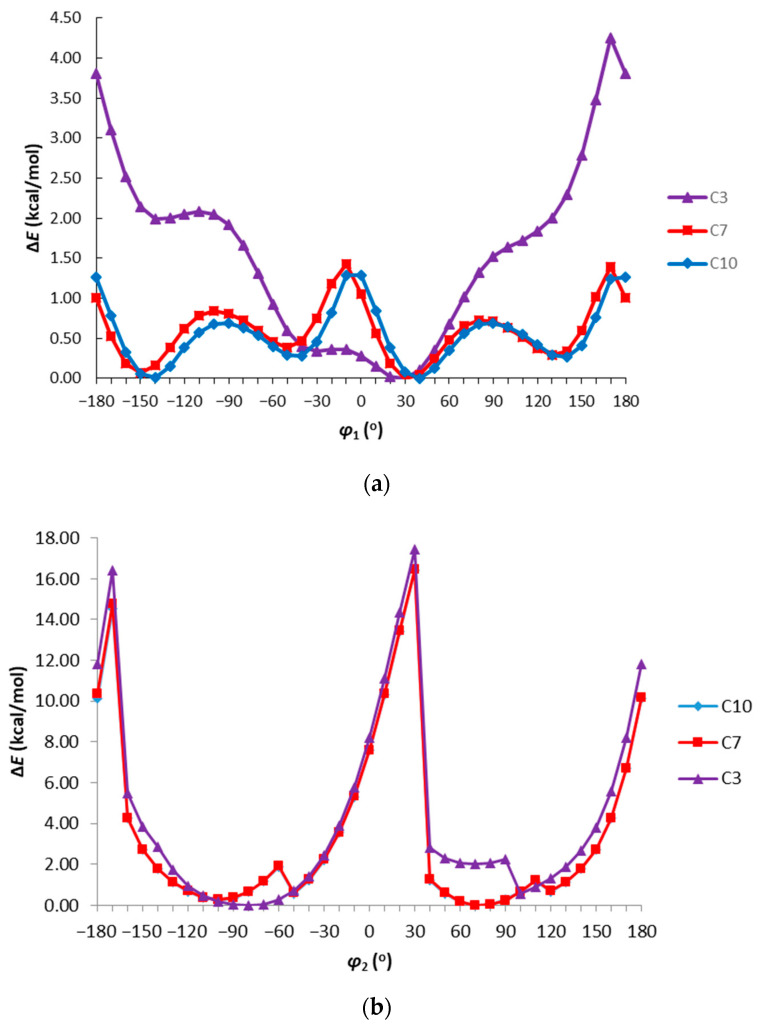
The energy effect of the free rotation for the torsion angle (**a**) *φ*_1_ = N4–C3–C61–N(C)62 and (**b**) *φ*_2_ = C3–N4–C41–C42 calculated for C3, C7, and C10 using the semiempirical AM1 method.

The investigated triazole molecules with pyridil substituents have two rotational degrees of freedom on the single bonds connecting the substituents with the 1,2,4-triazole ring. The conformational preferences of the analyzed molecules were characterized by the calculation of the energy effect of the free-rotation at these bonds for the model compounds C3, C7, and C10 using the AM1 method. The molecules of these compounds have the pyridil-2yl, pyridyl-3-yl, and pyridil-4-yl substituent in C3, C7, and C10, respectively, and the same 2,4-dichlorophenyl group at the 1,2,4-triazole ring. The energies of conformations were minimized, and all geometrical parameters optimized for each rotation with a 10° increment from −180 to 180° of *φ*_1_ = N4–C3–C61–N(C)62 and *φ*_2_ = C3–N4–C41–C42 torsion angles (Figure 2). 

As can be seen in Figure 2a, in the case of the torsion angle *φ*_1_, the energy barrier is small and is 4.5 kcal/mol for the pyridil-2-yl substituent at C3 and 1.5 kcal/mol for the pyridil-3(4)-yl substituents at C7 and C10, respectively. It can be seen that the height of the energy barrier depends on the position of the nitrogen atom in the pyridine ring and the highest energy barrier is observed for a pyridil-2-yl substituent. However, calculated energy barriers for the pyridil substituent indicate the possibility of free rotation of this group in physiological conditions. In all cases, four minima of energy are visible for *φ*_1_ of ± 30 and ± 150°, which is consistent with the values of this torsion angle observed for C1 and C12 in the crystalline state. In the case of the torsion angle *φ*_2_ (Figure 2b), the energy distribution and the height of the energy barrier do not depend on the position of the nitrogen atom in the pyridine ring. The height of the energy barrier of about 18 kcal/mol may indicate the possibility of inhibition of rotation on the bond under physiological conditions. Energy minima are observed for the mutually perpendicular positions of the benzene and triazole rings, which is consistent with the data obtained from X-ray analysis.

The values of theoretically calculated logP (octanol-water partition coefficient) (Table 2) are in the narrow range, except for C6, from 4.63 for C12 to 5.91 for C8 and C10. The clearly different value of logP of 0.97 for C6 results from the presence of polar-hydrophilic NO_2_ nitro group in its molecule. The positive values of logP show that all investigated compounds exhibit a rather strong affinity to the organic phase.

The view of the molecules C1–C14 in conformation obtained after energy minimization and geometrical parameters optimization at DFT/B3LYP/6-311++G(d,p) level with the vectors of dipole moments is shown in Figure S2 (Supplementary Materials). The dipole moments, NBO atomic net charges, energies of HOMO, and LUMO orbitals calculated for C1–C14 are presented in Table 2. 

It can be seen that the molecules are polar with a maximum dipole moment of 6.515 D for C6 and relatively larger values of dipole moments are observed for compounds with a pyridine-2-yl substituent at the 1,2,4-triazole ring. For most molecules, the dipole moment vector is directed from the benzene to the pyridine ring. The NBO net charge on nitrogen and sulfur atoms in common fragments of the molecular structures of C1–C14 changes in narrow ranges and practically does not depend on the substituents on the 1,2,4-triazole ring. The negative charge in absolute value on these heteroatoms changes according to the relationship: N4 > N (pyridines) > N1 > N2 > S5.

The energy values of the frontier orbitals HOMO and LUMO and the energy gap between these orbitals are presented in Table 2. The energy of HOMO orbitals are similar for all analyzed molecules and change in a relatively narrow energy range of 12.02 kcal/mol. Although the larger energy differences are observed for the LUMO orbital of 41.07 kcal/mol and energy gap of 24.07 kcal/mol, however, these values may indicate similar chemical reactivity and stability of molecules C1–C14. 

**Figure 3:**
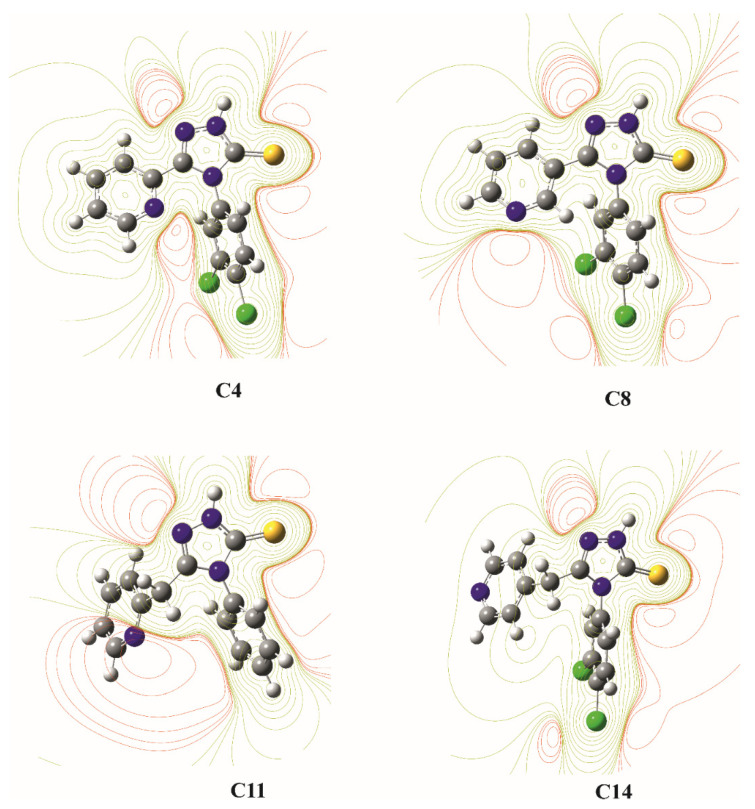
The contour maps of molecular electrostatic potential for compounds C4, C8, C11, and C14 in the plane of the 1,2,4-triazole ring; the red lines represent regions with the negative electrostatic potential and yellow lines represent regions with the positive electrostatic potential.

The molecular electrostatic potential (MEP) distribution in the plane of the 1,2,4-triazole ring for the most active against *Mycobacterium tuberculosis* compounds C4, C8, C11, and C14 is shown in Figure 3. For the remaining analyzed compound, the MEP distribution is presented in Figure S3 (Supplementary Materials). The MEP distribution in 3-(pyridin-2-yl(methyl))-1,2,4-triazoline-5-thione part of molecules is very similar for all investigated compounds. In this area, clear minima of the MEP are observed in the immediate vicinity of the nitrogen and sulfur atoms with large negative net charge as potential sites of electrophilic attack. Moreover, the local minima of MEP are also located near the chlorine (C3, C4, C7, C8, C10, C13, C14) and oxygen (C6, C12) atoms in the substituents at the *para* position of the phenyl ring. The MEP distribution as correlated with the partial charge on the atoms shows that the amino N1 atom can act as a hydrogen bond donor (positive electrostatic potential), while the S and other N atoms in 3-(pyridin-2-ylmethyl)-1,2,4-triazolin-5-thione system can act as hydrogen bond acceptors (negative electrostatic potential). These preferences are fully compatible with hydrogen bonds observed in the crystals of C1, C12, and C13 and indicate potential active sites on the investigated molecules for interactions with a molecular target related to their biological activity.

### 3.4. Antimycobacterial Activity

All obtained pyridine-1,2,4-triazoles were tested in vitro against *M. tuberculosis* H_37_Ra*, M. phlei, M. smegmatis,* and *M. timereck.* The antimicrobial activity was determined on the basis of the observed zones of growth inhibition (mm) of selected bacterial strains in the presence of the tested compounds and by determining the minimum inhibitory concentration (MIC) of growth of individual mycobacteria strains for each of the tested substances. After analyzing the data obtained by the disc diffusion method, it was found that the C1–C14 derivatives showed differential anti-tuberculosis potential. The largest zones of growth inhibition against the tested microorganisms were observed for the compound C11 (34–35.9 mm) and C4 (21.5–26.9 mm), regardless of the strain used. Satisfactory values were also obtained for the compounds C8 (21.9–24.7 mm) and C9 (10.9–19.6 mm). The anti-tuberculosis activity of the remaining compounds depended both on the structure and type of strains. Taking into account the type of strain, the highest values of the zone of inhibition of growth were observed for 2-pyridine substituted 1,2,4-triazole derivatives against all tested strains. Detailed data is provided in Table 3.

The next stage of in vitro studies was to determine the minimum inhibitory concentration (MIC) of microorganisms against the tested substances (Table 4). The obtained results clearly confirmed the antituberculosis potential of compound C4, which turned out to be the most active against *Mycobacterium* H_37_Ra (MIC = 0.976 μg/mL), *Mycobaterium pheli* (MIC = 7.81 μg/mL) and *Mycobacerium timereck* (62.6 μg/mL). Satisfactory results were obtained with compounds C8, C11, C14 versus *Myc.* H_37_Ra*, Myc. pheli, Myc. timereck* (MIC = 31.25–62.5 μg/mL). Interestingly, none of the compounds showed promising activity against *Myc. smegmatis*. Having analyzed the structures of the tested pyridine-triazole derivatives, it can be assumed that the presence of the 3,4-dichlorophenyl substituent (C4, C8, C14) determines the anti-tuberculosis activity. Interestingly, the repositioning of chlorine in the ring results in loss of activity (C3). Moreover, the presence of a 2-substituted pyridine ring increases the activity against the strains tested. Only in the case of *Myc. smegmatis*, the minimum inhibitory concentration for all compounds tested is greater than 125 µg/mL.

### 3.5. Molecular Docking

The next stage of the research was molecular docking of investigated 1,2,4-triazoles **C1**–**C14** to a selected molecular target related to their antibacterial activity against *Mycobacterium tuberculosis*. The crystal structure of the cytochrome P450 CYP121 of *Mycobacterium tuberculosis*, obtained from expression system *Escherichia coli* in complex with 1-[(4-chlorophenyl)methyl]-4-(3-imidazol-1-ylpropyl)piperazin-2-one (9KE) and heme as a prosthetic group (Figure S4; Supplementary Materials) obtained from Protein Data Bank (PDB ID: 5O4K), was selected as a molecular target. Literature reports that CYP121 catalyzes the reaction between two tyrosine residues of cyclodityrosine substrate in *Mycobacterium Tuberculosis* and it is essential for the viability of the bacterium [41,47]. Moreover, the molecular docking of 1,2,4-triazole derivatives with antimycobacterial potency to CYP121 are known [48], therefore the P450 CYP121 was chosen for the molecular docking simulation. 

The molecular docking study showed that the molecules of all investigated 1,24-triazoles can bind to the active site of the P450 CYP121 enzyme. The values of the ChemPLP scoring function and the interactions between ligands and residues of amino acids in the active site are presented in Table 5. 

**Figure 4:**
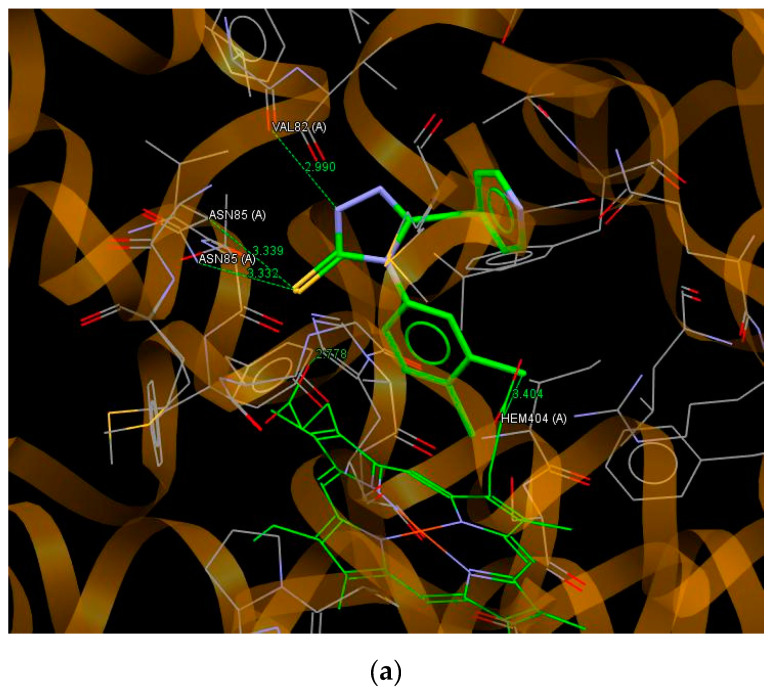

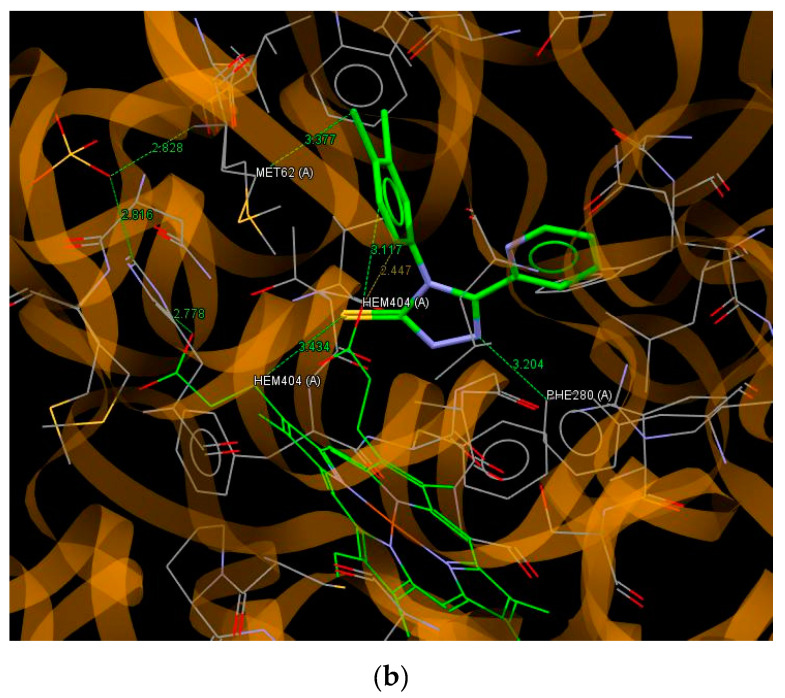
A view of the interaction between C14 (**a**) C4 and (**b**) and the binding site of cytochrome P450.

It can be seen that the values of the scoring function ChemPLP for all ligands are in a relatively narrow range. The highest value of ChemPLP of 68.29 is obtained for compound C14, while the lowest value of 53.67 is observed for compound C8. For the most active compound C4, the scoring function has a value of 57.68. Relatively higher values of ChemPLP are observed for pyridilmethyl derivatives containing a methylene linker between the 1,2,4-triazole and pyridine ring. Almost all ligands bind to the active site of cytochrome P450 via S5… C hydrogen bonds with MET62 and the heme molecule. The nitrogen atoms of the 1,2,4-triazole (N1 and N2) and pyridine ring play an important role in the interaction with the active site of cytochrome P450 through hydrogen bonds with the residues of VAL82, PHE280, ARG386, SER237, ASN85, and MET62. The interactions of the best fitted to the binding site of P450 CYP121 compound C14 are shown in Figure 4a. This ligand binds to the active site by hydrogen bonds N1… O (VAL82), S5… N(ASN85), S5… C(ASN85) and Cl43… C(HEME). The most active against *Mycobacterium Tuberculosis* compound C4 is bound to the binding pocket of cytochrome P450 enzyme via hydrogen bonds N2…C(PHE280), Cl44…C(MET62) and S5… C, C45… O and C46… O with the heme molecule (Figure 4b). 

It is worth noting, that the conformation of molecule C4 observed in the enzyme binding site is very similar to that of a molecule C1 in a crystal state with the torsion angles describing the position of the pyridine and phenyl substituent in relation to the 1,2,4-triazole ring N4–C3–C61–N62 of 18.5° (−16.7(4)° in C1) and C3–N4–C41–C42 of 90.1° (88.8(3)° in C1). Moreover, the conformation in the binding pose of C4, like the conformation of C14, is close to their starting conformations for the docking process obtained as a result of energy minimization and energy optimization at the DFT level. The comparison of these conformations is shown in Figure S5 (Supplementary Materials). The observed slight differences in conformation for C14 result from the flexibility of the pyridin-4-ylmethyl substituent with two rotational degrees of freedom.

## 4. Conclusions

The synthesized 1,2,4-triazoles were obtained in the cyclization reaction of appropriate thiosemicarbazide derivatives in an alkaline medium. The structures of obtained compounds were confirmed by spectroscopic methods and X-ray investigations. The X-ray analysis performed for C1, C12 and C13 confirmed the synthesis pathway, the assumed molecular structures of the analyzed 1,2,4-triazole derivatives, and showed that all investigated compounds exist in N1-amino/S5-thione tautomeric form in the crystalline state. The preferences for the formation of hydrogen bonds in the crystal are consistent with the tautomeric form observed in the crystalline state and with the distribution of the molecular electrostatic potential. Theoretical calculations at DFT/B3LYP/6-311++G(d,p) level showed a large similarity of conformational preferences and a similar electronic structure of investigated molecules, which can indicate their similar reactivity and stability in a physiological environment. The results of the molecular docking of the tested 1,2,4-triazoles to the cytochrome P450 CYP121 exhibited that the best fitting to the active site of the enzyme was shown by compound C14 with noticeable activity against *Mycobacterial tuberculosis* Myc.H_37_Ra within in vitro tests. The most active in biological tests compound **C4** shows large values of the scoring function ChemPLP and binds to the active site of the enzyme via hydrogen bonds to the amino acid residue of phenylalanine and methionine and to the heme molecule. The sulfur and nitrogen atoms of the 1,2,4-triazol-5-thione system and nitrogen atom of the pyridine ring play a key role in the interaction with the active site of cytochrome P450 CYP121. It should be noted that the molecules of all investigated 1,24-triazoles can bind to the active site of the P450 CYP121 enzyme.

## Figures and Tables

**Table 1:** Hydrogen-bond geometry (Å, °).

D–H…A	D–H	H…A	D…A	D–H…A
**C1**				
N1–H1…S5*^i^*	0.89(3)	2.37(4)	3.261(2)	175(3)
*i* = −*x*, 2 − *y*, −*z*				
**C12**				
N1–H1…N63*^i^*	0.92(3)	1.88(3)	2.789(3)	171(2)
*i* = *x*, 3/2 − *y*, −1/2 + *z*				
**C13**				
N1–H1…N63*^i^*	0.99(3)	1.83(3)	2.815(4)	170(3)
C43–H43…N2*^ii^*	0.93	2.53	3.435(4)	164
C46–H46…S5*^iii^*	0.93	2.84	3.675(4)	150
C65–H65…S5*^iv^*	0.93	2.87	3.714(4)	152
C31–H312…S*^v^*	0.97	2.87	3.552(4)	129
*i* = −1 + *x*, 1 + *y*, *z*; *ii* = *x*, −1+*y*, *z*; *iii* = −*x*, 1 − *y*, 1 − *z*; *iv* = −*x*, 1 − *y*, −*z*; *v* = 1 + *x*, *y*, *z*				

**Table 2:** The dipole moments, *D_m_* (D), NBO atomic charges, (*e*), energies of HOMO and LUMO orbitals, *E_HOMO_* and *E_LUMO_* (kcal/mol), Δ*E* = *E_LUMO_*-*E_HOMO_* (kcal/mol) calculated for isolated molecules at DFT/B3LYP/6-311++G(d,p) level and calculated log*P* coefficient for C1–C14.

Compound	*D_m_*	NBO Charge					*E_HOMO_*	*E_LUMO_*	Δ*E*	log*P*
		N1	N2	N4	N*_pyridil_*	S				
**C1**	5.631	−0.381	−0.274	−0.455	−0.455	−0.207	−134.17	−45.45	88.68	4.95
**C2**	5.736	−0.381	−0.276	−0.462	−0.451	−0.205	−133.80	−45.42	88.38	5.33
**C3**	6.001	−0.389	−0.273	−0.460	−0.451	−0.200	−136.58	−48.19	88.39	5.85
**C4**	6.262	−0.382	−0.274	−0.462	−0.451	−0.202	−137.66	−49.22	88.44	5.85
**C5**	3.265	−0.384	−0.277	−0.471	−0.444	−0.206	−136.88	−43.70	93.18	5.40
**C6**	6.515	−0.382	−0.270	−0.471	−0.440	−0.193	−144.86	−73.52	77.33	0.97
**C7**	3.502	−0.384	−0.276	−0.474	−0.443	−0.202	−139.61	−46.19	93.42	5.81
**C8**	3.901	−0.384	−0.275	−0.465	−0.441	−0.206	−140.92	−47.72	93.20	5.91
**C9**	2.870	−0.382	−0.258	−0.455	−0.442	−0.196	−138.88	−49.32	89.55	5.02
**C10**	1.587	−0.381	−0.265	−0.472	−0.439	−0.206	−141.92	−51.26	90.65	5.91
**C11**	4.538	−0.394	−0.308	−0.476	−0.469	−0.222	−132.84	−32.45	100.39	4.75
**C12**	3.208	−0.384	−0.281	−0.461	−0.444	−0.217	−133.86	−42.17	91.68	4.63
**C13**	3.694	−0.393	−0.294	−0.482	−0.444	−0.205	−138.47	−38.39	99.94	5.85
**C14**	4.688	−0.392	−0.291	−0.480	−0.448	−0.205	−140.64	−39.24	101.40	5.85

**Table 3:** Inhibition zone for tested compounds (250 μg of the compound was used per well). The results were expressed as mean value ± standard deviation (SD).

Inhibition Zone (mm) 250 μg of Compound PerWell						
	Ar	R	Mycobacterium H_37_Ra	Mycobacterium *Phlei*	Mycobacterium *Smegmatis*	Mycobacterium *Timereck*
**C1**	pyridin-2-yl	2-FC_6_H_4_	15.1 ± 0.61	15.1 ± 0.53	16.0 ± 0.81	15.1 ± 0.67
**C2**	pyridin-2-yl	2-ClC_6_H_4_	19.6 ± 0.43	21.5 ± 1.12	17.2 ± 0.98	15.3 ± 0.84
**C3**	pyridin-2-yl	2,4-Cl_2_C_6_H_3_	10.2 ± 0.8	17.0 ± 0.78	17.9 ± 0.77	20.1 ± 1.15
**C4**	pyridin-2-yl	3,4-Cl_2_C_6_H_3_	26.9 ± 0.78	22.7 ± 0.91	22.3 ± 1.05	21.5 ± 0.9
**C5**	pyridin-3-yl	2-ClC_6_H_4_	12.3 ± 0.78	0.0	10.2 ± 0.86	16.5 ± 0.41
**C6**	pyridin-3-yl	4-NO_2_C_6_H_4_	0.0	0.0	0.0	10.6 ± 0.53
**C7**	pyridin-3-yl	2,4-Cl_2_C_6_H_3_	0.0	8.9 ± 0.66	0.0	0.0
**C8**	pyridin-3-yl	3,4-Cl_2_C_6_H_3_	22.2 ± 0.72	21.9 ± 0.29	23.1 ± 0.6	24.7 ± 0.87
**C9**	pyridin-4-yl	2-FC_6_H_4_	10.6 ± 0.53	10.9 ± 0.66	12.3 ± 0.92	19.6 ± 0.53
**C10**	pyridin-4-yl	2,4-Cl_2_C_6_H_3_	0.0	0.0	0.0	0.0
**C11**	pyridin-2-yl	C_6_H_5_	35.9 ± 0.49	34.0 ± 0.93	35.3 ± 0.7	35.1 ± 0.58
**C12**	pyridin-3-yl	4-CH_3_OC_6_H_4_	10.6 ± 0.85	0.0	0.0	0.0
**C13**	pyridin-3-ylmethyl	2,4-Cl_2_C_6_H_3_	12.0 ± 0.67	0.0	0.0	0.0
**C14**	pyridin-4-ylmethyl	3,4-Cl_2_C_6_H_3_	20.7 ± 0.29	0.0	18.2 ± 0.53	18.6 ± 0.98

**Table 4:** Minimal inhibitory concentration for compounds **C1–C14**.

MIC (µg/mL)						
	Ar	R	Mycobacterium H_37_Ra	Mycobacterium *Phlei*	Mycobacterium *Smegmatis*	Mycobacterium *Timereck*
**C1**	pyridin-2-yl	2-FC_6_H_4_	62.5	500	>500	>500
**C4**	pyridin-2-yl	3,4-Cl_2_C_6_H_3_	0.976	7.81	125	62.5
**C8**	pyridin-3-yl	3,4-Cl_2_C_6_H_3_	62.5	31.25	125	62.5
**C11**	pyridin-2-yl	C_6_H_5_	62.5	31.25	250	62.5
**C14**	pyridin-2-ylmethyl	3,4-Cl_2_C_6_H_3_	62.5	125	>500	125

Compounds C1, C2, C5-C7, C9, C10, C12, and C13 MIC ≥ 125 μg/mL.

**Table 5:** The results of the molecular docking of ligands **C1**–**C14** to the P450 CYP121.

Compound	*f_CHEMPLP_*	Ligand—Amino Acids Interactions
**C1**	56.67	F42…C(HEME); S5…C(MET62); N1…O(HEME); N2…C(HEME)
**C2**	57.79	N1…O(HEME); N2…C(HEME); S5…C(MET62)
**C3**	54.59	S5…C(HEME); N1…O(VAL82)
**C4**	57.68	N2…C(PHE280); S5…C(HEME); C45…O(HEME); C46…O(HEME); Cl44…(MET62)
**C5**	57.04	S5…C(MET62); N2…C(PHE280)
**C6**	67.83	S5…C(MET62) × 2; N63…N(ARG386); O…O(SER237)
**C7**	55.14	S5…C(MET62); N63…C(PHE280); Cl44…(C(PHE280); Cl42…O(HEME)
**C8**	53.67	N63…N(ARG386); Cl43…C(VAL78)
**C9**	58.71	S5…C((MET62); N64…O(SER237)
**C10**	60.20	N2…C(HEME); N64…N(ARG386); C63…O(SER237); C43…O(HEME)
**C11**	65.77	S5…C(MET62) × 2; S5…C(HEME)
**C12**	55.89	N1…O(VAL82)
**C13**	66.94	S5…N(ASN85); S5…C(ASN85); N64…C(MET62); N1…O(VAL82); N2…C(MET62)
**C14**	68.29	N1…O(VAL82); S5…N(ASN85); S5…C(ASN85); Cl43…C(HEME)

## Supplementary Materials

Supplementary materials are available online.
