# Supplementary material for: New Application of 1,2,4-Triazole Derivatives as Antitubercular Agents. Structure, In Vitro Screening and Docking Studies
Source: Molecules. 2020 Dec 19;25(24):6033. doi: 10.3390/molecules25246033 (PMC7767103; doi:10.3390/molecules25246033)

Supplementary material for publication on line

New application of 1,2,4-triazole derivatives as antitubercular agents. Structure, in vitro screening and docking studies.

Zbigniew Karczmarzyk[1], Marta Swatko-Ossor[2], Waldemar Wysocki[2], Monika Drozd[3], Grazyna Ginalska[2], Anna Pachut-Stec[3], Monika Pitucha[3*]

[1]Faculty of Science, Siedlce University of Natural Sciences and Humanities, Siedlce, Poland, [2]Department of Biochemistry and Biotechnology, Faculty of Pharmacy, Medical University of Lublin, Poland, [3]Independent Radiopharmacy Unit, Faculty of Pharmacy, Medical University of Lublin, Poland; monika.pitucha@umlub.pl (M.P)

* Correspondence: monika.pitucha@umlub.pl (M.P); Tel.: +48-81-448-74-20

X-ray structure determination

CIF for C1

```
data_shelx

_audit_creation_method            'SHELXL-2014/7'
_shelx_SHELXL_version_number      '2014/7'
_chemical_name_systematic
;
4-(2-fluorophenyl)-3-(2-pyridyl)-1H-1,2,4-triazole-5-thione
;
_chemical_name_common             ?
_chemical_melting_point           ?
_chemical_formula_moiety          'C13 H9 F N4 S'
_chemical_formula_sum             'C13 H9 F N4 S'
_chemical_formula_weight          272.30

loop_
 _atom_type_symbol
 _atom_type_description
 _atom_type_scat_dispersion_real
 _atom_type_scat_dispersion_imag
 _atom_type_scat_source
 'C'  'C'   0.0033   0.0016
 'International Tables Vol C Tables 4.2.6.8 and 6.1.1.4'
 'H'  'H'   0.0000   0.0000
 'International Tables Vol C Tables 4.2.6.8 and 6.1.1.4'
 'N'  'N'   0.0061   0.0033
 'International Tables Vol C Tables 4.2.6.8 and 6.1.1.4'
 'F'  'F'   0.0171   0.0103
```

```
 'International Tables Vol C Tables 4.2.6.8 and 6.1.1.4'
 'S'  'S'   0.1246   0.1234
 'International Tables Vol C Tables 4.2.6.8 and 6.1.1.4'

_space_group_crystal_system       triclinic
_space_group_IT_number            2
_space_group_name_H-M_alt         'P -1'
_space_group_name_Hall            '-P 1'

_shelx_space_group_comment
;
The symmetry employed for this shelxl refinement is uniquely defined
by the following loop, which should always be used as a source of
symmetry information in preference to the above space-group names.
They are only intended as comments.
;

loop_
 _space_group_symop_operation_xyz
 'x, y, z'
 '-x, -y, -z'

_cell_length_a                  7.0908(6)
_cell_length_b                  7.8326(7)
_cell_length_c                  12.4147(10)
_cell_angle_alpha               71.722(8)
_cell_angle_beta                74.428(7)
_cell_angle_gamma               87.846(7)
_cell_volume                    629.87(10)
_cell_formula_units_Z           2
_cell_measurement_temperature     296(2)
_cell_measurement_reflns_used     1379
_cell_measurement_theta_min       1.8080
_cell_measurement_theta_max       28.0210

_exptl_crystal_description        prism
_exptl_crystal_colour             colourles
_exptl_crystal_density_meas       ?
_exptl_crystal_density_method     ?
_exptl_crystal_density_diffrn     1.436
_exptl_crystal_F_000              280
_exptl_transmission_factor_min    ?
_exptl_transmission_factor_max    ?
_exptl_crystal_size_max           0.20
_exptl_crystal_size_mid           0.15
_exptl_crystal_size_min           0.10
_exptl_absorpt_coefficient_mu     0.259
_shelx_estimated_absorpt_T_min    ?
_shelx_estimated_absorpt_T_max    ?
_exptl_absorpt_correction_T_min   0.79631
_exptl_absorpt_correction_T_max   1.00000
```

_exptl_absorpt_correction_type   'multi-scan'
_exptl_absorpt_process_details
;
CrysAlisPro, Agilent Technologies,
Version 1.171.37.35h (release 09-02-2015 CrysAlis171 .NET)
(compiled Feb 9 2015,16:26:32)
Empirical absorption correction using spherical harmonics,
 implemented in SCALE3 ABSPACK scaling algorithm.
;
_exptl_absorpt_special_details   ?
_diffrn_ambient_temperature   296(2)
_diffrn_radiation_wavelength   0.71073
_diffrn_radiation_type   MoK\a
_diffrn_source   ?
_diffrn_measurement_device_type   'KM4 CCD four-circle diffractometer'
_diffrn_measurement_method   '\w scans'
_diffrn_detector_area_resol_mean ?
_diffrn_reflns_number   4337
_diffrn_reflns_av_unetI/netI   0.0534
_diffrn_reflns_av_R_equivalents   0.0301
_diffrn_reflns_limit_h_min   -9
_diffrn_reflns_limit_h_max   8
_diffrn_reflns_limit_k_min   -9
_diffrn_reflns_limit_k_max   8
_diffrn_reflns_limit_l_min   -16
_diffrn_reflns_limit_l_max   16
_diffrn_reflns_theta_min   1.794
_diffrn_reflns_theta_max   29.112
_diffrn_reflns_theta_full   25.242
_diffrn_measured_fraction_theta_max   0.841
_diffrn_measured_fraction_theta_full 1.000
_diffrn_reflns_Laue_measured_fraction_max   0.841
_diffrn_reflns_Laue_measured_fraction_full   1.000
_diffrn_reflns_point_group_measured_fraction_max   0.841
_diffrn_reflns_point_group_measured_fraction_full 1.000
_reflns_number_total   2845
_reflns_number_gt   2092
_reflns_threshold_expression   'I > 2\s(I)'
_reflns_Friedel_coverage   0.000
_reflns_Friedel_fraction_max   .
_reflns_Friedel_fraction_full   .

_reflns_special_details
;
 Reflections were merged by SHELXL according to the crystal
 class for the calculation of statistics and refinement.

 _reflns_Friedel_fraction is defined as the number of unique
 Friedel pairs measured divided by the number that would be
 possible theoretically, ignoring centric projections and
 systematic absences.

;

_computing_data_collection
;
CrysAlisPro, Agilent Technologies,
Version 1.171.37.35h (release 09-02-2015 CrysAlis171 .NET)
(compiled Feb 9 2015,16:26:32)
;
_computing_cell_refinement
;
CrysAlisPro, Agilent Technologies,
Version 1.171.37.35h (release 09-02-2015 CrysAlis171 .NET)
(compiled Feb 9 2015,16:26:32)
;
_computing_data_reduction
;
CrysAlisPro, Agilent Technologies,
Version 1.171.37.35h (release 09-02-2015 CrysAlis171 .NET)
(compiled Feb 9 2015,16:26:32)
;
_computing_structure_solution   'SHELXS-2013/1 (Sheldrick, 2013)'
_computing_structure_refinement   'SHELXL-2014/7 (Sheldrick, 2014)'
_computing_molecular_graphics   'ORTEP3 for Windows (Farrugia, 2012)'
_computing_publication_material
;
SHELXL-2014/7 (Sheldrick, 2014) and WINGX (Farrugia, 2012)
;
_refine_special_details   ?
_refine_ls_structure_factor_coef Fsqd
_refine_ls_matrix_type   full
_refine_ls_weighting_scheme   calc
_refine_ls_weighting_details
'w=1/[\s^2^(Fo^2^)+(0.0698P)^2^+0.1997P] where P=(Fo^2^+2Fc^2^)/3'
_atom_sites_solution_primary   refxyz
_atom_sites_solution_secondary   ?
_atom_sites_solution_hydrogens   mixed
_refine_ls_hydrogen_treatment   mixed
_refine_ls_extinction_method   none
_refine_ls_extinction_coef   .
_refine_ls_number_reflns   2845
_refine_ls_number_parameters   175
_refine_ls_number_restraints   0
_refine_ls_R_factor_all   0.0802
_refine_ls_R_factor_gt   0.0559
_refine_ls_wR_factor_ref   0.1638
_refine_ls_wR_factor_gt   0.1416
_refine_ls_goodness_of_fit_ref   1.080
_refine_ls_restrained_S_all   1.080
_refine_ls_shift/su_max   0.000
_refine_ls_shift/su_mean   0.000

loop_
 _atom_site_label
 _atom_site_type_symbol
 _atom_site_fract_x
 _atom_site_fract_y
 _atom_site_fract_z
 _atom_site_U_iso_or_equiv
 _atom_site_adp_type
 _atom_site_occupancy
 _atom_site_site_symmetry_order
 _atom_site_calc_flag
 _atom_site_refinement_flags_posn
 _atom_site_refinement_flags_adp
 _atom_site_refinement_flags_occupancy
 _atom_site_disorder_assembly
 _atom_site_disorder_group
S5 S 1.02937(11) -0.08188(11) 0.83396(6) 0.0529(3) Uani 1 1 d . . . . .
F42 F 0.8709(3) 0.2988(3) 0.56558(16) 0.0712(6) Uani 1 1 d . . . . .
N1 N 0.7815(4) 0.1184(3) 0.9481(2) 0.0489(6) Uani 1 1 d . . . . .
H1 H 0.835(5) 0.101(4) 1.008(3) 0.073 Uiso 1 1 d . U . . .
N2 N 0.6229(4) 0.2223(3) 0.9409(2) 0.0514(6) Uani 1 1 d . . . . .
N4 N 0.7062(3) 0.1032(3) 0.79626(18) 0.0397(5) Uani 1 1 d . . . . .
N62 N 0.4161(4) 0.3240(3) 0.6953(2) 0.0544(6) Uani 1 1 d . . . . .
C3 C 0.5785(4) 0.2111(4) 0.8478(2) 0.0427(6) Uani 1 1 d . . . . .
C5 C 0.8395(4) 0.0452(4) 0.8613(2) 0.0417(6) Uani 1 1 d . . . . .
C41 C 0.6988(4) 0.0385(3) 0.7014(2) 0.0391(6) Uani 1 1 d . . . . .
C42 C 0.7849(4) 0.1372(4) 0.5867(2) 0.0430(6) Uani 1 1 d . . . . .
C43 C 0.7865(4) 0.0760(4) 0.4945(3) 0.0548(8) Uani 1 1 d . . . . .
H43 H 0.8438 0.1460 0.4171 0.082 Uiso 1 1 calc R U . . .
C44 C 0.7012(5) -0.0912(5) 0.5192(3) 0.0616(9) Uani 1 1 d . . . . .
H44 H 0.7010 -0.1353 0.4579 0.092 Uiso 1 1 calc R U . . .
C45 C 0.6160(5) -0.1947(4) 0.6338(3) 0.0665(9) Uani 1 1 d . . . . .
H45 H 0.5590 -0.3078 0.6491 0.100 Uiso 1 1 calc R U . . .
C46 C 0.6146(5) -0.1310(4) 0.7268(3) 0.0554(7) Uani 1 1 d . . . . .
H46 H 0.5584 -0.2009 0.8044 0.083 Uiso 1 1 calc R U . . .
C61 C 0.4153(4) 0.3050(4) 0.8062(2) 0.0448(6) Uani 1 1 d . . . . .
C63 C 0.2672(5) 0.4080(4) 0.6589(3) 0.0639(9) Uani 1 1 d . . . . .
H63 H 0.2657 0.4234 0.5817 0.096 Uiso 1 1 calc R U . . .
C64 C 0.1161(5) 0.4733(4) 0.7273(3) 0.0637(9) Uani 1 1 d . . . . .
H64 H 0.0134 0.5279 0.6981 0.096 Uiso 1 1 calc R U . . .
C65 C 0.1199(5) 0.4563(5) 0.8396(3) 0.0678(9) Uani 1 1 d . . . . .
H65 H 0.0214 0.5023 0.8876 0.102 Uiso 1 1 calc R U . . .
C66 C 0.2721(5) 0.3696(4) 0.8810(3) 0.0605(8) Uani 1 1 d . . . . .
H66 H 0.2776 0.3554 0.9573 0.091 Uiso 1 1 calc R U . . .

loop_
 _atom_site_aniso_label
 _atom_site_aniso_U_11
 _atom_site_aniso_U_22
 _atom_site_aniso_U_33
 _atom_site_aniso_U_23

_atom_site_aniso_U_13
 _atom_site_aniso_U_12
S5 0.0579(5) 0.0677(5) 0.0436(4) -0.0256(4) -0.0237(3) 0.0197(4)
F42 0.0875(13) 0.0660(12) 0.0552(11) -0.0081(9) -0.0214(10) -0.0176(10)
N1 0.0581(14) 0.0573(15) 0.0399(12) -0.0215(11) -0.0215(11) 0.0130(11)
N2 0.0593(14) 0.0574(15) 0.0458(13) -0.0239(12) -0.0202(11) 0.0128(12)
N4 0.0463(12) 0.0420(12) 0.0346(11) -0.0135(9) -0.0159(9) 0.0051(9)
N62 0.0650(15) 0.0557(15) 0.0575(15) -0.0277(13) -0.0316(12) 0.0217(12)
C3 0.0493(15) 0.0431(14) 0.0388(13) -0.0161(12) -0.0138(11) 0.0059(11)
C5 0.0487(15) 0.0442(14) 0.0348(12) -0.0120(11) -0.0160(11) 0.0013(11)
C41 0.0434(14) 0.0432(14) 0.0371(13) -0.0159(11) -0.0178(11) 0.0052(11)
C42 0.0433(14) 0.0470(15) 0.0445(14) -0.0173(12) -0.0179(11) 0.0019(11)
C43 0.0518(16) 0.076(2) 0.0438(16) -0.0249(16) -0.0192(13) 0.0100(15)
C44 0.069(2) 0.076(2) 0.066(2) -0.0434(19) -0.0372(17) 0.0188(17)
C45 0.080(2) 0.0518(19) 0.086(3) -0.0317(19) -0.041(2) 0.0018(16)
C46 0.0645(18) 0.0487(17) 0.0587(18) -0.0169(15) -0.0251(15) -0.0021(14)
C61 0.0499(15) 0.0393(14) 0.0474(15) -0.0150(12) -0.0158(12) 0.0052(11)
C63 0.076(2) 0.059(2) 0.078(2) -0.0320(18) -0.0447(19) 0.0209(16)
C64 0.0592(19) 0.0496(18) 0.087(2) -0.0177(17) -0.0331(18) 0.0141(14)
C65 0.060(2) 0.059(2) 0.076(2) -0.0184(18) -0.0090(17) 0.0178(15)
C66 0.065(2) 0.0601(19) 0.0514(17) -0.0155(15) -0.0126(15) 0.0167(15)

_geom_special_details
;
 All esds (except the esd in the dihedral angle between two l.s. planes)
 are estimated using the full covariance matrix. The cell esds are taken
 into account individually in the estimation of esds in distances, angles
 and torsion angles; correlations between esds in cell parameters are only
 used when they are defined by crystal symmetry. An approximate (isotropic)
 treatment of cell esds is used for estimating esds involving l.s. planes.
;

loop_
 _geom_bond_atom_site_label_1
 _geom_bond_atom_site_label_2
 _geom_bond_distance
 _geom_bond_site_symmetry_2
 _geom_bond_publ_flag
S5 C5 1.669(3) . ?
F42 C42 1.345(3) . ?
N1 C5 1.339(3) . ?
N1 N2 1.370(3) . ?
N1 H1 0.89(3) . ?
N2 C3 1.305(3) . ?
N4 C5 1.377(3) . ?
N4 C3 1.383(3) . ?
N4 C41 1.434(3) . ?
N62 C63 1.330(4) . ?
N62 C61 1.336(3) . ?
C3 C61 1.472(4) . ?
C41 C42 1.370(4) . ?

C41 C46 1.384(4) . ?
C42 C43 1.371(4) . ?
C43 C44 1.372(4) . ?
C43 H43 0.9300 . ?
C44 C45 1.378(5) . ?
C44 H44 0.9300 . ?
C45 C46 1.392(4) . ?
C45 H45 0.9300 . ?
C46 H46 0.9300 . ?
C61 C66 1.378(4) . ?
C63 C64 1.369(5) . ?
C63 H63 0.9300 . ?
C64 C65 1.365(5) . ?
C64 H64 0.9300 . ?
C65 C66 1.384(5) . ?
C65 H65 0.9300 . ?
C66 H66 0.9300 . ?

loop_
 _geom_angle_atom_site_label_1
 _geom_angle_atom_site_label_2
 _geom_angle_atom_site_label_3
 _geom_angle
 _geom_angle_site_symmetry_1
 _geom_angle_site_symmetry_3
 _geom_angle_publ_flag
C5 N1 N2 113.7(2) . . ?
C5 N1 H1 125(2) . . ?
N2 N1 H1 121(2) . . ?
C3 N2 N1 104.4(2) . . ?
C5 N4 C3 108.0(2) . . ?
C5 N4 C41 122.4(2) . . ?
C3 N4 C41 129.3(2) . . ?
C63 N62 C61 116.5(3) . . ?
N2 C3 N4 110.5(2) . . ?
N2 C3 C61 122.8(2) . . ?
N4 C3 C61 126.7(2) . . ?
N1 C5 N4 103.3(2) . . ?
N1 C5 S5 129.3(2) . . ?
N4 C5 S5 127.35(19) . . ?
C42 C41 C46 119.7(2) . . ?
C42 C41 N4 120.9(2) . . ?
C46 C41 N4 119.3(2) . . ?
F42 C42 C43 119.7(3) . . ?
F42 C42 C41 118.1(2) . . ?
C43 C42 C41 122.2(2) . . ?
C42 C43 C44 118.2(3) . . ?
C42 C43 H43 120.9 . . ?
C44 C43 H43 120.9 . . ?
C43 C44 C45 120.8(3) . . ?
C43 C44 H44 119.6 . . ?

C45 C44 H44 119.6 . . ?
C44 C45 C46 120.5(3) . . ?
C44 C45 H45 119.8 . . ?
C46 C45 H45 119.8 . . ?
C41 C46 C45 118.5(3) . . ?
C41 C46 H46 120.7 . . ?
C45 C46 H46 120.7 . . ?
N62 C61 C66 123.4(3) . . ?
N62 C61 C3 117.0(2) . . ?
C66 C61 C3 119.6(3) . . ?
N62 C63 C64 124.4(3) . . ?
N62 C63 H63 117.8 . . ?
C64 C63 H63 117.8 . . ?
C65 C64 C63 118.3(3) . . ?
C65 C64 H64 120.9 . . ?
C63 C64 H64 120.9 . . ?
C64 C65 C66 119.1(3) . . ?
C64 C65 H65 120.4 . . ?
C66 C65 H65 120.4 . . ?
C61 C66 C65 118.2(3) . . ?
C61 C66 H66 120.9 . . ?
C65 C66 H66 120.9 . . ?

loop_
 _geom_torsion_atom_site_label_1
 _geom_torsion_atom_site_label_2
 _geom_torsion_atom_site_label_3
 _geom_torsion_atom_site_label_4
 _geom_torsion
 _geom_torsion_site_symmetry_1
 _geom_torsion_site_symmetry_2
 _geom_torsion_site_symmetry_3
 _geom_torsion_site_symmetry_4
 _geom_torsion_publ_flag
C5 N1 N2 C3 1.1(3) . . . . ?
N1 N2 C3 N4 -0.3(3) . . . . ?
N1 N2 C3 C61 -179.5(2) . . . . ?
C5 N4 C3 N2 -0.6(3) . . . . ?
C41 N4 C3 N2 172.4(2) . . . . ?
C5 N4 C3 C61 178.6(3) . . . . ?
C41 N4 C3 C61 -8.5(4) . . . . ?
N2 N1 C5 N4 -1.4(3) . . . . ?
N2 N1 C5 S5 177.3(2) . . . . ?
C3 N4 C5 N1 1.2(3) . . . . ?
C41 N4 C5 N1 -172.4(2) . . . . ?
C3 N4 C5 S5 -177.6(2) . . . . ?
C41 N4 C5 S5 8.9(4) . . . . ?
C5 N4 C41 C42 -99.2(3) . . . . ?
C3 N4 C41 C42 88.8(3) . . . . ?
C5 N4 C41 C46 76.6(3) . . . . ?
C3 N4 C41 C46 -95.4(3) . . . . ?

C46 C41 C42 F42 -177.9(2) . . . . ?
N4 C41 C42 F42 -2.1(4) . . . . ?
C46 C41 C42 C43 1.8(4) . . . . ?
N4 C41 C42 C43 177.6(2) . . . . ?
F42 C42 C43 C44 178.6(3) . . . . ?
C41 C42 C43 C44 -1.1(4) . . . . ?
C42 C43 C44 C45 0.1(5) . . . . ?
C43 C44 C45 C46 0.1(5) . . . . ?
C42 C41 C46 C45 -1.5(4) . . . . ?
N4 C41 C46 C45 -177.4(3) . . . . ?
C44 C45 C46 C41 0.6(5) . . . . ?
C63 N62 C61 C66 -1.0(4) . . . . ?
C63 N62 C61 C3 179.6(3) . . . . ?
N2 C3 C61 N62 162.3(3) . . . . ?
N4 C3 C61 N62 -16.7(4) . . . . ?
N2 C3 C61 C66 -17.2(4) . . . . ?
N4 C3 C61 C66 163.8(3) . . . . ?
C61 N62 C63 C64 -0.5(5) . . . . ?
N62 C63 C64 C65 2.0(5) . . . . ?
C63 C64 C65 C66 -1.9(5) . . . . ?
N62 C61 C66 C65 1.0(5) . . . . ?
C3 C61 C66 C65 -179.6(3) . . . . ?
C64 C65 C66 C61 0.5(5) . . . . ?

_refine_diff_density_max   0.697
_refine_diff_density_min   -0.255
_refine_diff_density_rms   0.055

_shelx_res_file
;

    shelx.res created by SHELXL-2014/7

TITL t1 in P-1
CELL 0.71073   7.0908   7.8326 12.4147   71.722   74.428   87.846
ZERR   2.00   0.0006   0.0007   0.0010   0.008   0.007   0.007
LATT   1
SFAC C   H   N   F   S
UNIT 26 18 8 2 2
MERG   2
FMAP   2
PLAN   -2
ACTA
BOND $H
CONF
L.S. 40
WGHT   0.069800   0.199700
FVAR   3.49246
MOLE   1
S5   5   1.029375   -0.081877   0.833960   11.00000   0.05789   0.06767 =

```
        0.04357   -0.02557   -0.02374    0.01970
F42   4    0.870932    0.298845    0.565578    11.00000    0.08746    0.06599 =
        0.05521   -0.00812   -0.02138   -0.01763
N1    3    0.781519    0.118388    0.948119    11.00000    0.05813    0.05730 =
        0.03990   -0.02147   -0.02150    0.01295
H1    2    0.835365    0.100618    1.007969    11.00000   -1.50000
N2    3    0.622858    0.222322    0.940877    11.00000    0.05927    0.05741 =
        0.04583   -0.02388   -0.02017    0.01279
N4    3    0.706198    0.103198    0.796261    11.00000    0.04626    0.04200 =
        0.03462   -0.01348   -0.01588    0.00509
N62   3    0.416052    0.323985    0.695349    11.00000    0.06496    0.05571 =
        0.05748   -0.02774   -0.03159    0.02165
C3    1    0.578480    0.211067    0.847799    11.00000    0.04933    0.04307 =
        0.03878   -0.01606   -0.01376    0.00594
C5    1    0.839460    0.045242    0.861251    11.00000    0.04872    0.04419 =
        0.03475   -0.01199   -0.01596    0.00125
C41   1    0.698826    0.038488    0.701409    11.00000    0.04342    0.04316 =
        0.03708   -0.01593   -0.01783    0.00519
C42   1    0.784935    0.137151    0.586726    11.00000    0.04326    0.04699 =
        0.04447   -0.01733   -0.01788    0.00195
C43   1    0.786498    0.076002    0.494457    11.00000    0.05178    0.07646 =
        0.04378   -0.02492   -0.01922    0.00999
AFIX  43
H43   2    0.843831    0.146004    0.417109    11.00000   -1.50000
AFIX   0
C44   1    0.701230   -0.091247    0.519222    11.00000    0.06868    0.07558 =
        0.06622   -0.04337   -0.03725    0.01883
AFIX  43
H44   2    0.700961   -0.135316    0.457875    11.00000   -1.50000
AFIX   0
C45   1    0.616026   -0.194675    0.633824    11.00000    0.08043    0.05177 =
        0.08609   -0.03173   -0.04134    0.00176
AFIX  43
H45   2    0.559036   -0.307825    0.649135    11.00000   -1.50000
AFIX   0
C46   1    0.614630   -0.131044    0.726837    11.00000    0.06452    0.04868 =
        0.05867   -0.01688   -0.02509   -0.00209
AFIX  43
H46   2    0.558361   -0.200904    0.804367    11.00000   -1.50000
AFIX   0
C61   1    0.415255    0.305046    0.806229    11.00000    0.04988    0.03930 =
        0.04744   -0.01497   -0.01579    0.00522
C63   1    0.267226    0.408015    0.658871    11.00000    0.07601    0.05880 =
        0.07765   -0.03196   -0.04471    0.02090
AFIX  43
H63   2    0.265733    0.423433    0.581719    11.00000   -1.50000
AFIX   0
C64   1    0.116059    0.473342    0.727318    11.00000    0.05924    0.04958 =
        0.08681   -0.01773   -0.03308    0.01410
AFIX  43
H64   2    0.013450    0.527918    0.698085    11.00000   -1.50000
```

```
AFIX   0
C65   1    0.119857    0.456291    0.839576    11.00000    0.05961    0.05882 =
        0.07603   -0.01839   -0.00901    0.01785
AFIX  43
H65   2    0.021400    0.502327    0.887567    11.00000   -1.50000
AFIX   0
C66   1    0.272095    0.369626    0.881030    11.00000    0.06530    0.06014 =
        0.05142   -0.01555   -0.01259    0.01669
AFIX  43
H66   2    0.277633    0.355350    0.957347    11.00000   -1.50000
AFIX   0
HKLF    4

REM  t1 in P-1
REM R1 =  0.0559 for    2092 Fo > 4sig(Fo)  and  0.0802 for all    2845 data
REM    175 parameters refined using      0 restraints

END

WGHT      0.0651      0.2555

REM Highest difference peak  0.697,  deepest hole -0.255,  1-sigma level  0.055
Q1    1   0.3191  0.3773  0.9534  11.00000  0.05    0.20
Q2    1   0.4976  0.2649  0.8239  11.00000  0.05    0.19
;
```

CIF for C12

```
data_shelx

_audit_creation_method            'SHELXL-2014/7'
_shelx_SHELXL_version_number      '2014/7'
_chemical_name_systematic
;
4-(4-methoxyphenyl)-3-(3-pyridyl)-1H-1,2,4-triazole-5-thione
;
_chemical_name_common             ?
_chemical_melting_point           ?
_chemical_formula_moiety          'C14 H12 N4 O S'
_chemical_formula_sum             'C14 H12 N4 O S'
_chemical_formula_weight          284.34

loop_
 _atom_type_symbol
 _atom_type_description
 _atom_type_scat_dispersion_real
 _atom_type_scat_dispersion_imag
 _atom_type_scat_source
 'C'  'C'   0.0033   0.0016
 'International Tables Vol C Tables 4.2.6.8 and 6.1.1.4'
 'H'  'H'   0.0000   0.0000
```

```
 'International Tables Vol C Tables 4.2.6.8 and 6.1.1.4'
 'N'  'N'   0.0061   0.0033
 'International Tables Vol C Tables 4.2.6.8 and 6.1.1.4'
 'O'  'O'   0.0106   0.0060
 'International Tables Vol C Tables 4.2.6.8 and 6.1.1.4'
 'S'  'S'   0.1246   0.1234
 'International Tables Vol C Tables 4.2.6.8 and 6.1.1.4'

_space_group_crystal_system       monoclinic
_space_group_IT_number            14
_space_group_name_H-M_alt         'P 21/c'
_space_group_name_Hall            '-P 2ybc'

_shelx_space_group_comment
;
The symmetry employed for this shelxl refinement is uniquely defined
by the following loop, which should always be used as a source of
symmetry information in preference to the above space-group names.
They are only intended as comments.
;

loop_
 _space_group_symop_operation_xyz
 'x, y, z'
 '-x, y+1/2, -z+1/2'
 '-x, -y, -z'
 'x, -y-1/2, z-1/2'

_cell_length_a                    9.3878(19)
_cell_length_b                    9.858(2)
_cell_length_c                    14.600(3)
_cell_angle_alpha                 90
_cell_angle_beta                  90.59(3)
_cell_angle_gamma                 90
_cell_volume                      1351.1(5)
_cell_formula_units_Z             4
_cell_measurement_temperature     296(2)
_cell_measurement_reflns_used     2157
_cell_measurement_theta_min       2.4820
_cell_measurement_theta_max       27.8000

_exptl_crystal_description        needle
_exptl_crystal_colour             colourles
_exptl_crystal_density_meas       ?
_exptl_crystal_density_method     ?
_exptl_crystal_density_diffrn     1.398
_exptl_crystal_F_000              592
_exptl_transmission_factor_min    ?
_exptl_transmission_factor_max    ?
_exptl_crystal_size_max           0.30
_exptl_crystal_size_mid           0.10
```

_exptl_crystal_size_min   0.10
_exptl_absorpt_coefficient_mu   0.240
_shelx_estimated_absorpt_T_min   ?
_shelx_estimated_absorpt_T_max   ?
_exptl_absorpt_correction_T_min   0.91656
_exptl_absorpt_correction_T_max   1.00000
_exptl_absorpt_correction_type   'multi-scan'
_exptl_absorpt_process_details
;
CrysAlisPro, Agilent Technologies,
Version 1.171.37.35h (release 09-02-2015 CrysAlis171 .NET)
(compiled Feb 9 2015,16:26:32)
Empirical absorption correction using spherical harmonics,
 implemented in SCALE3 ABSPACK scaling algorithm.
;
_exptl_absorpt_special_details   ?
_diffrn_ambient_temperature   296(2)
_diffrn_radiation_wavelength   0.71073
_diffrn_radiation_type   MoK\a
_diffrn_source   ?
_diffrn_measurement_device_type   'KM4 CCD four-circle diffractometer'
_diffrn_measurement_method   '\w scans'
_diffrn_detector_area_resol_mean ?
_diffrn_reflns_number   5862
_diffrn_reflns_av_unetI/netI   0.0442
_diffrn_reflns_av_R_equivalents   0.0307
_diffrn_reflns_limit_h_min   -12
_diffrn_reflns_limit_h_max   12
_diffrn_reflns_limit_k_min   -7
_diffrn_reflns_limit_k_max   12
_diffrn_reflns_limit_l_min   -8
_diffrn_reflns_limit_l_max   19
_diffrn_reflns_theta_min   2.170
_diffrn_reflns_theta_max   29.234
_diffrn_reflns_theta_full   25.242
_diffrn_measured_fraction_theta_max   0.837
_diffrn_measured_fraction_theta_full 1.000
_diffrn_reflns_Laue_measured_fraction_max   0.837
_diffrn_reflns_Laue_measured_fraction_full   1.000
_diffrn_reflns_point_group_measured_fraction_max   0.837
_diffrn_reflns_point_group_measured_fraction_full 1.000
_reflns_number_total   3073
_reflns_number_gt   2286
_reflns_threshold_expression   'I > 2\s(I)'
_reflns_Friedel_coverage   0.000
_reflns_Friedel_fraction_max   .
_reflns_Friedel_fraction_full   .

_reflns_special_details
;
 Reflections were merged by SHELXL according to the crystal

class for the calculation of statistics and refinement.

_reflns_Friedel_fraction is defined as the number of unique
Friedel pairs measured divided by the number that would be
possible theoretically, ignoring centric projections and
systematic absences.
;

_computing_data_collection
;
CrysAlisPro, Agilent Technologies,
Version 1.171.37.35h (release 09-02-2015 CrysAlis171 .NET)
(compiled Feb 9 2015,16:26:32)
;
_computing_cell_refinement
;
CrysAlisPro, Agilent Technologies,
Version 1.171.37.35h (release 09-02-2015 CrysAlis171 .NET)
(compiled Feb 9 2015,16:26:32)
;
_computing_data_reduction
;
CrysAlisPro, Agilent Technologies,
Version 1.171.37.35h (release 09-02-2015 CrysAlis171 .NET)
(compiled Feb 9 2015,16:26:32)
;
_computing_structure_solution 'SHELXS-2013/1 (Sheldrick, 2013)'
_computing_structure_refinement 'SHELXL-2014/7 (Sheldrick, 2014)'
_computing_molecular_graphics 'ORTEP3 for Windows (Farrugia, 2012)'
_computing_publication_material
;
SHELXL-2014/7 (Sheldrick, 2014) and WINGX (Farrugia, 2012)
;
_refine_special_details ?
_refine_ls_structure_factor_coef Fsqd
_refine_ls_matrix_type full
_refine_ls_weighting_scheme calc
_refine_ls_weighting_details
'w=1/[\s^2^(Fo^2^)+(0.0479P)^2^+0.4399P] where P=(Fo^2^+2Fc^2^)/3'
_atom_sites_solution_primary refxyz
_atom_sites_solution_secondary ?
_atom_sites_solution_hydrogens mixed
_refine_ls_hydrogen_treatment mixed
_refine_ls_extinction_method none
_refine_ls_extinction_coef .
_refine_ls_number_reflns 3073
_refine_ls_number_parameters 184
_refine_ls_number_restraints 0
_refine_ls_R_factor_all 0.0661
_refine_ls_R_factor_gt 0.0423
_refine_ls_wR_factor_ref 0.1259

_refine_ls_wR_factor_gt 0.0958
_refine_ls_goodness_of_fit_ref 1.113
_refine_ls_restrained_S_all 1.113
_refine_ls_shift/su_max 0.000
_refine_ls_shift/su_mean 0.000

loop_
 _atom_site_label
 _atom_site_type_symbol
 _atom_site_fract_x
 _atom_site_fract_y
 _atom_site_fract_z
 _atom_site_U_iso_or_equiv
 _atom_site_adp_type
 _atom_site_occupancy
 _atom_site_site_symmetry_order
 _atom_site_calc_flag
 _atom_site_refinement_flags_posn
 _atom_site_refinement_flags_adp
 _atom_site_refinement_flags_occupancy
 _atom_site_disorder_assembly
 _atom_site_disorder_group
S5 S 0.24115(7) 0.56484(6) 0.16270(4) 0.04008(19) Uani 1 1 d . . . . .
O47 O -0.0266(2) 0.99272(19) -0.14262(14) 0.0608(5) Uani 1 1 d . . . . .
N1 N 0.3579(2) 0.37414(18) 0.05440(12) 0.0333(4) Uani 1 1 d . . . . .
H1 H 0.369(3) 0.310(3) 0.0997(17) 0.050 Uiso 1 1 d . U . . .
N2 N 0.3863(2) 0.34250(17) -0.03527(12) 0.0339(4) Uani 1 1 d . . . . .
N4 N 0.27969(18) 0.54222(16) -0.02347(11) 0.0281(4) Uani 1 1 d . . . . .
N63 N 0.3873(2) 0.34019(18) -0.32216(12) 0.0375(4) Uani 1 1 d . . . . .
C3 C 0.3377(2) 0.44692(19) -0.08155(13) 0.0280(4) Uani 1 1 d . . . . .
C5 C 0.2930(2) 0.4941(2) 0.06514(14) 0.0293(4) Uani 1 1 d . . . . .
C41 C 0.2016(2) 0.6621(2) -0.05053(13) 0.0291(4) Uani 1 1 d . . . . .
C42 C 0.2628(2) 0.7883(2) -0.04163(14) 0.0345(5) Uani 1 1 d . . . . .
H42 H 0.3530 0.7971 -0.0153 0.052 Uiso 1 1 calc R U . . .
C43 C 0.1897(3) 0.9026(2) -0.07196(16) 0.0405(5) Uani 1 1 d . . . . .
H43 H 0.2306 0.9881 -0.0660 0.061 Uiso 1 1 calc R U . . .
C44 C 0.0556(3) 0.8883(2) -0.11120(16) 0.0408(6) Uani 1 1 d . . . . .
C45 C -0.0042(3) 0.7602(3) -0.11995(17) 0.0459(6) Uani 1 1 d . . . . .
H45 H -0.0940 0.7507 -0.1466 0.069 Uiso 1 1 calc R U . . .
C46 C 0.0678(2) 0.6474(2) -0.08960(16) 0.0397(5) Uani 1 1 d . . . . .
H46 H 0.0269 0.5618 -0.0953 0.060 Uiso 1 1 calc R U . . .
C48 C 0.0307(3) 1.1257(3) -0.1398(2) 0.0671(9) Uani 1 1 d . . . . .
H481 H 0.1109 1.1311 -0.1799 0.101 Uiso 1 1 calc R U . . .
H482 H 0.0607 1.1466 -0.0783 0.101 Uiso 1 1 calc R U . . .
H483 H -0.0408 1.1894 -0.1592 0.101 Uiso 1 1 calc R U . . .
C61 C 0.3524(2) 0.45924(19) -0.18097(13) 0.0280(4) Uani 1 1 d . . . . .
C62 C 0.3647(2) 0.3412(2) -0.23250(14) 0.0338(5) Uani 1 1 d . . . . .
H62 H 0.3565 0.2584 -0.2025 0.051 Uiso 1 1 calc R U . . .
C64 C 0.3957(3) 0.4603(2) -0.36444(15) 0.0404(6) Uani 1 1 d . . . . .
H64 H 0.4110 0.4612 -0.4273 0.061 Uiso 1 1 calc R U . . .
C65 C 0.3828(3) 0.5820(2) -0.32007(15) 0.0415(6) Uani 1 1 d . . . . .

H65 H 0.3886 0.6632 -0.3523 0.062 Uiso 1 1 calc R U . . .
C66 C 0.3611(3) 0.5820(2) -0.22687(15) 0.0361(5) Uani 1 1 d . . . . .
H66 H 0.3523 0.6634 -0.1952 0.054 Uiso 1 1 calc R U . . .

loop_
 _atom_site_aniso_label
 _atom_site_aniso_U_11
 _atom_site_aniso_U_22
 _atom_site_aniso_U_33
 _atom_site_aniso_U_23
 _atom_site_aniso_U_13
 _atom_site_aniso_U_12
S5 0.0534(4) 0.0410(3) 0.0260(3) -0.0038(2) 0.0077(2) -0.0020(3)
O47 0.0589(12) 0.0503(11) 0.0732(14) 0.0149(10) -0.0022(10) 0.0226(10)
N1 0.0471(11) 0.0299(9) 0.0229(9) 0.0053(7) 0.0032(8) 0.0020(8)
N2 0.0459(11) 0.0290(9) 0.0269(9) 0.0028(7) 0.0043(8) 0.0035(8)
N4 0.0357(9) 0.0265(9) 0.0221(8) 0.0001(7) 0.0016(7) 0.0021(7)
N63 0.0511(12) 0.0339(10) 0.0276(9) -0.0050(8) 0.0004(8) 0.0008(9)
C3 0.0341(11) 0.0251(10) 0.0248(10) -0.0010(8) 0.0016(8) -0.0004(8)
C5 0.0333(11) 0.0280(10) 0.0265(10) 0.0022(8) 0.0013(8) -0.0039(9)
C41 0.0356(11) 0.0287(10) 0.0230(9) 0.0000(8) 0.0027(8) 0.0034(9)
C42 0.0390(12) 0.0321(11) 0.0325(11) -0.0023(9) -0.0036(9) 0.0001(10)
C43 0.0506(14) 0.0302(12) 0.0409(13) -0.0005(10) 0.0001(11) 0.0032(10)
C44 0.0442(13) 0.0419(13) 0.0364(12) 0.0049(10) 0.0043(10) 0.0153(11)
C45 0.0349(12) 0.0517(15) 0.0509(15) 0.0034(12) -0.0058(11) 0.0044(11)
C46 0.0358(12) 0.0388(12) 0.0445(13) -0.0012(10) -0.0012(10) -0.0034(10)
C48 0.086(2) 0.0478(17) 0.068(2) 0.0187(14) 0.0159(16) 0.0275(16)
C61 0.0335(11) 0.0274(10) 0.0231(10) -0.0007(8) 0.0006(8) -0.0003(9)
C62 0.0441(12) 0.0280(11) 0.0293(11) 0.0001(9) 0.0014(9) -0.0001(10)
C64 0.0555(15) 0.0424(13) 0.0233(10) -0.0025(9) 0.0023(10) 0.0020(11)
C65 0.0632(16) 0.0321(12) 0.0293(11) 0.0037(9) 0.0036(10) 0.0013(11)
C66 0.0544(14) 0.0268(11) 0.0273(11) -0.0018(8) 0.0021(10) 0.0008(10)

_geom_special_details
;
 All esds (except the esd in the dihedral angle between two l.s. planes)
 are estimated using the full covariance matrix. The cell esds are taken
 into account individually in the estimation of esds in distances, angles
 and torsion angles; correlations between esds in cell parameters are only
 used when they are defined by crystal symmetry. An approximate (isotropic)
 treatment of cell esds is used for estimating esds involving l.s. planes.
;

loop_
 _geom_bond_atom_site_label_1
 _geom_bond_atom_site_label_2
 _geom_bond_distance
 _geom_bond_site_symmetry_2
 _geom_bond_publ_flag
S5 C5 1.663(2) . ?
O47 C44 1.363(3) . ?

O47 C48 1.417(4) . ?
N1 C5 1.340(3) . ?
N1 N2 1.375(2) . ?
N1 H1 0.92(3) . ?
N2 C3 1.311(2) . ?
N4 C3 1.381(2) . ?
N4 C5 1.383(2) . ?
N4 C41 1.443(2) . ?
N63 C62 1.328(3) . ?
N63 C64 1.338(3) . ?
C3 C61 1.465(3) . ?
C41 C42 1.376(3) . ?
C41 C46 1.382(3) . ?
C42 C43 1.389(3) . ?
C42 H42 0.9300 . ?
C43 C44 1.385(3) . ?
C43 H43 0.9300 . ?
C44 C45 1.387(3) . ?
C45 C46 1.373(3) . ?
C45 H45 0.9300 . ?
C46 H46 0.9300 . ?
C48 H481 0.9600 . ?
C48 H482 0.9600 . ?
C48 H483 0.9600 . ?
C61 C66 1.386(3) . ?
C61 C62 1.391(3) . ?
C62 H62 0.9300 . ?
C64 C65 1.370(3) . ?
C64 H64 0.9300 . ?
C65 C66 1.378(3) . ?
C65 H65 0.9300 . ?
C66 H66 0.9300 . ?

loop_
 _geom_angle_atom_site_label_1
 _geom_angle_atom_site_label_2
 _geom_angle_atom_site_label_3
 _geom_angle
 _geom_angle_site_symmetry_1
 _geom_angle_site_symmetry_3
 _geom_angle_publ_flag
C44 O47 C48 118.4(2) . . ?
C5 N1 N2 113.87(17) . . ?
C5 N1 H1 125.0(16) . . ?
N2 N1 H1 120.3(16) . . ?
C3 N2 N1 104.05(16) . . ?
C3 N4 C5 107.99(16) . . ?
C3 N4 C41 126.23(16) . . ?
C5 N4 C41 125.29(16) . . ?
C62 N63 C64 117.30(18) . . ?
N2 C3 N4 110.79(17) . . ?

N2 C3 C61 122.69(18) . . ?
N4 C3 C61 126.44(17) . . ?
N1 C5 N4 103.30(17) . . ?
N1 C5 S5 127.42(16) . . ?
N4 C5 S5 129.29(16) . . ?
C42 C41 C46 120.7(2) . . ?
C42 C41 N4 120.29(18) . . ?
C46 C41 N4 118.93(19) . . ?
C41 C42 C43 119.9(2) . . ?
C41 C42 H42 120.0 . . ?
C43 C42 H42 120.0 . . ?
C44 C43 C42 119.6(2) . . ?
C44 C43 H43 120.2 . . ?
C42 C43 H43 120.2 . . ?
O47 C44 C43 124.9(2) . . ?
O47 C44 C45 115.4(2) . . ?
C43 C44 C45 119.7(2) . . ?
C46 C45 C44 120.7(2) . . ?
C46 C45 H45 119.6 . . ?
C44 C45 H45 119.6 . . ?
C45 C46 C41 119.4(2) . . ?
C45 C46 H46 120.3 . . ?
C41 C46 H46 120.3 . . ?
O47 C48 H481 109.5 . . ?
O47 C48 H482 109.5 . . ?
H481 C48 H482 109.5 . . ?
O47 C48 H483 109.5 . . ?
H481 C48 H483 109.5 . . ?
H482 C48 H483 109.5 . . ?
C66 C61 C62 117.59(19) . . ?
C66 C61 C3 123.93(18) . . ?
C62 C61 C3 118.41(18) . . ?
N63 C62 C61 123.65(19) . . ?
N63 C62 H62 118.2 . . ?
C61 C62 H62 118.2 . . ?
N63 C64 C65 123.5(2) . . ?
N63 C64 H64 118.3 . . ?
C65 C64 H64 118.3 . . ?
C64 C65 C66 118.8(2) . . ?
C64 C65 H65 120.6 . . ?
C66 C65 H65 120.6 . . ?
C65 C66 C61 119.19(19) . . ?
C65 C66 H66 120.4 . . ?
C61 C66 H66 120.4 . . ?

loop_
 _geom_torsion_atom_site_label_1
 _geom_torsion_atom_site_label_2
 _geom_torsion_atom_site_label_3
 _geom_torsion_atom_site_label_4
 _geom_torsion

_geom_torsion_site_symmetry_1
_geom_torsion_site_symmetry_2
_geom_torsion_site_symmetry_3
_geom_torsion_site_symmetry_4
_geom_torsion_publ_flag
C5 N1 N2 C3 0.3(2) . . . . ?
N1 N2 C3 N4 -0.1(2) . . . . ?
N1 N2 C3 C61 176.84(18) . . . . ?
C5 N4 C3 N2 0.0(2) . . . . ?
C41 N4 C3 N2 -172.29(18) . . . . ?
C5 N4 C3 C61 -176.85(19) . . . . ?
C41 N4 C3 C61 10.9(3) . . . . ?
N2 N1 C5 N4 -0.3(2) . . . . ?
N2 N1 C5 S5 179.40(16) . . . . ?
C3 N4 C5 N1 0.2(2) . . . . ?
C41 N4 C5 N1 172.53(18) . . . . ?
C3 N4 C5 S5 -179.49(16) . . . . ?
C41 N4 C5 S5 -7.1(3) . . . . ?
C3 N4 C41 C42 -107.4(2) . . . . ?
C5 N4 C41 C42 81.6(3) . . . . ?
C3 N4 C41 C46 69.4(3) . . . . ?
C5 N4 C41 C46 -101.6(2) . . . . ?
C46 C41 C42 C43 0.1(3) . . . . ?
N4 C41 C42 C43 176.85(19) . . . . ?
C41 C42 C43 C44 -0.1(3) . . . . ?
C48 O47 C44 C43 3.4(4) . . . . ?
C48 O47 C44 C45 -177.0(2) . . . . ?
C42 C43 C44 O47 179.4(2) . . . . ?
C42 C43 C44 C45 -0.1(4) . . . . ?
O47 C44 C45 C46 -179.2(2) . . . . ?
C43 C44 C45 C46 0.4(4) . . . . ?
C44 C45 C46 C41 -0.4(4) . . . . ?
C42 C41 C46 C45 0.2(3) . . . . ?
N4 C41 C46 C45 -176.6(2) . . . . ?
N2 C3 C61 C66 -151.1(2) . . . . ?
N4 C3 C61 C66 25.4(3) . . . . ?
N2 C3 C61 C62 26.0(3) . . . . ?
N4 C3 C61 C62 -157.5(2) . . . . ?
C64 N63 C62 C61 -1.4(3) . . . . ?
C66 C61 C62 N63 1.6(3) . . . . ?
C3 C61 C62 N63 -175.7(2) . . . . ?
C62 N63 C64 C65 0.3(4) . . . . ?
N63 C64 C65 C66 0.5(4) . . . . ?
C64 C65 C66 C61 -0.2(4) . . . . ?
C62 C61 C66 C65 -0.8(3) . . . . ?
C3 C61 C66 C65 176.4(2) . . . . ?

_refine_diff_density_max 0.206
_refine_diff_density_min -0.252
_refine_diff_density_rms 0.055

```
_shelx_res_file
;

    shelx.res created by SHELXL-2014/7

TITL T15 in P2(1)/c
CELL  0.71073   9.3878   9.8576  14.6003   90.000   90.586   90.000
ZERR    4.00   0.0019   0.0020   0.0029    0.000    0.030    0.000
LATT   1
SYMM  - X, 1/2 + Y, 1/2 - Z
SFAC  C    H    N    O    S
UNIT  56 48 16 4 4
MERG   2
FMAP   2
PLAN   -2
ACTA
BOND   $H
CONF
L.S.  40
WGHT    0.047900    0.439900
FVAR       1.59215
MOLE    1
S5    5    0.241152    0.564845    0.162700    11.00000    0.05339    0.04096 =
        0.02604   -0.00377    0.00773   -0.00204
O47   4   -0.026586    0.992715   -0.142623    11.00000    0.05889    0.05028 =
        0.07318    0.01492   -0.00225    0.02259
N1    3    0.357904    0.374142    0.054397    11.00000    0.04710    0.02993 =
        0.02291    0.00527    0.00323    0.00204
H1    2    0.369350    0.309782    0.099680    11.00000   -1.50000
N2    3    0.386303    0.342496   -0.035267    11.00000    0.04595    0.02904 =
        0.02690    0.00284    0.00432    0.00353
N4    3    0.279690    0.542218   -0.023475    11.00000    0.03566    0.02649 =
        0.02208    0.00014    0.00163    0.00210
N63   3    0.387341    0.340187   -0.322163    11.00000    0.05106    0.03387 =
        0.02761   -0.00500    0.00038    0.00076
C3    1    0.337663    0.446921   -0.081553    11.00000    0.03413    0.02508 =
        0.02476   -0.00100    0.00160   -0.00037
C5    1    0.292966    0.494086    0.065143    11.00000    0.03331    0.02804 =
        0.02653    0.00218    0.00134   -0.00394
C41   1    0.201632    0.662100   -0.050534    11.00000    0.03558    0.02868 =
        0.02301    0.00005    0.00271    0.00335
C42   1    0.262786    0.788329   -0.041629    11.00000    0.03895    0.03207 =
        0.03247   -0.00233   -0.00359    0.00007
AFIX  43
H42   2    0.352954    0.797135   -0.015326    11.00000   -1.50000
AFIX   0
C43   1    0.189724    0.902553   -0.071958    11.00000    0.05058    0.03017 =
        0.04085   -0.00051    0.00005    0.00319
AFIX  43
H43   2    0.230592    0.988077   -0.065969    11.00000   -1.50000
AFIX   0
```

```
C44   1    0.055558    0.888310   -0.111198    11.00000    0.04418    0.04186 =
         0.03640    0.00489    0.00432    0.01531
C45   1   -0.004195    0.760236   -0.119947    11.00000    0.03493    0.05173 =
         0.05091    0.00339   -0.00576    0.00438
AFIX  43
H45   2   -0.094001    0.750701   -0.146650    11.00000   -1.50000
AFIX   0
C46   1    0.067824    0.647373   -0.089604    11.00000    0.03575    0.03879 =
         0.04446   -0.00124   -0.00119   -0.00336
AFIX  43
H46   2    0.026942    0.561813   -0.095292    11.00000   -1.50000
AFIX   0
C48   1    0.030683    1.125674   -0.139791    11.00000    0.08629    0.04775 =
         0.06765    0.01866    0.01585    0.02747
AFIX 133
H481  2    0.110898    1.131086   -0.179885    11.00000   -1.50000
H482  2    0.060705    1.146565   -0.078342    11.00000   -1.50000
H483  2   -0.040756    1.189446   -0.159228    11.00000   -1.50000
AFIX   0
C61   1    0.352409    0.459245   -0.180969    11.00000    0.03346    0.02739 =
         0.02313   -0.00070    0.00062   -0.00035
C62   1    0.364652    0.341192   -0.232504    11.00000    0.04409    0.02796 =
         0.02932    0.00007    0.00141   -0.00009
AFIX  43
H62   2    0.356498    0.258362   -0.202517    11.00000   -1.50000
AFIX   0
C64   1    0.395672    0.460308   -0.364436    11.00000    0.05551    0.04241 =
         0.02329   -0.00254    0.00229    0.00200
AFIX  43
H64   2    0.411014    0.461188   -0.427261    11.00000   -1.50000
AFIX   0
C65   1    0.382798    0.582040   -0.320073    11.00000    0.06324    0.03215 =
         0.02927    0.00372    0.00358    0.00126
AFIX  43
H65   2    0.388619    0.663233   -0.352256    11.00000   -1.50000
AFIX   0
C66   1    0.361063    0.582008   -0.226874    11.00000    0.05436    0.02684 =
         0.02725   -0.00178    0.00210    0.00077
AFIX  43
H66   2    0.352308    0.663354   -0.195163    11.00000   -1.50000
AFIX   0
HKLF    4

REM  T15 in P2(1)/c
REM R1 =  0.0423 for    2286 Fo > 4sig(Fo)  and  0.0661 for all    3073 data
REM    184 parameters refined using      0 restraints

END

WGHT      0.0547      0.2965
```

```
REM Highest difference peak  0.206,  deepest hole -0.252,  1-sigma level  0.055
Q1    1   0.0116  0.8229 -0.0904  11.00000  0.05    0.21
Q2    1   0.1945  0.9996 -0.0164  11.00000  0.05    0.19
;
```

CIF for C13

```
data_shelx

_audit_creation_method            'SHELXL-2014/7'
_shelx_SHELXL_version_number      '2014/7'
_chemical_name_systematic
;
4-(2,4-dichlorophenyl)-3-(4-pyridylmethyl)-1H-1,2,4-triazole-5-thione
;
_chemical_name_common             ?
_chemical_melting_point           ?
_chemical_formula_moiety          'C14 H10 Cl2 N4 S'
_chemical_formula_sum             'C14 H10 Cl2 N4 S'
_chemical_formula_weight          337.22

loop_
 _atom_type_symbol
 _atom_type_description
 _atom_type_scat_dispersion_real
 _atom_type_scat_dispersion_imag
 _atom_type_scat_source
 'C'  'C'   0.0033   0.0016
 'International Tables Vol C Tables 4.2.6.8 and 6.1.1.4'
 'H'  'H'   0.0000   0.0000
 'International Tables Vol C Tables 4.2.6.8 and 6.1.1.4'
 'N'  'N'   0.0061   0.0033
 'International Tables Vol C Tables 4.2.6.8 and 6.1.1.4'
 'S'  'S'   0.1246   0.1234
 'International Tables Vol C Tables 4.2.6.8 and 6.1.1.4'
 'Cl'  'Cl'   0.1484   0.1585
 'International Tables Vol C Tables 4.2.6.8 and 6.1.1.4'

_space_group_crystal_system       triclinic
_space_group_IT_number            2
_space_group_name_H-M_alt         'P -1'
_space_group_name_Hall            '-P 1'

_shelx_space_group_comment
;
The symmetry employed for this shelxl refinement is uniquely defined
by the following loop, which should always be used as a source of
symmetry information in preference to the above space-group names.
They are only intended as comments.
;
```

```
loop_
 _space_group_symop_operation_xyz
 'x, y, z'
 '-x, -y, -z'

_cell_length_a                    8.4234(10)
_cell_length_b                    8.8584(9)
_cell_length_c                    11.251(2)
_cell_angle_alpha                 81.356(13)
_cell_angle_beta                  83.534(12)
_cell_angle_gamma                 66.032(11)
_cell_volume                      757.21(19)
_cell_formula_units_Z             2
_cell_measurement_temperature     296(2)
_cell_measurement_reflns_used     1947
_cell_measurement_theta_min       1.8070
_cell_measurement_theta_max       26.5620

_exptl_crystal_description        block
_exptl_crystal_colour             colourles
_exptl_crystal_density_meas       ?
_exptl_crystal_density_method     ?
_exptl_crystal_density_diffrn     1.479
_exptl_crystal_F_000              344
_exptl_transmission_factor_min    ?
_exptl_transmission_factor_max    ?
_exptl_crystal_size_max           0.40
_exptl_crystal_size_mid           0.40
_exptl_crystal_size_min           0.10
_exptl_absorpt_coefficient_mu     0.564
_shelx_estimated_absorpt_T_min    ?
_shelx_estimated_absorpt_T_max    ?
_exptl_absorpt_correction_T_min   0.88389
_exptl_absorpt_correction_T_max   1.00000
_exptl_absorpt_correction_type    'multi-scan'
_exptl_absorpt_process_details
;
CrysAlisPro, Agilent Technologies,
Version 1.171.37.35h (release 09-02-2015 CrysAlis171 .NET)
(compiled Feb  9 2015,16:26:32)
Empirical absorption correction using spherical harmonics,
 implemented in SCALE3 ABSPACK scaling algorithm.
;
_exptl_absorpt_special_details    ?
_diffrn_ambient_temperature       296(2)
_diffrn_radiation_wavelength      0.71073
_diffrn_radiation_type            MoK\a
_diffrn_source                    ?
_diffrn_measurement_device_type   'KM4 CCD four-circle diffractometer'
_diffrn_measurement_method        '\w scans'
_diffrn_detector_area_resol_mean  ?
```

_diffrn_reflns_number 4996
_diffrn_reflns_av_unetI/netI 0.0628
_diffrn_reflns_av_R_equivalents ?
_diffrn_reflns_limit_h_min -11
_diffrn_reflns_limit_h_max 11
_diffrn_reflns_limit_k_min -11
_diffrn_reflns_limit_k_max 11
_diffrn_reflns_limit_l_min -14
_diffrn_reflns_limit_l_max 14
_diffrn_reflns_theta_min 1.834
_diffrn_reflns_theta_max 28.932
_diffrn_reflns_theta_full 25.242
_diffrn_measured_fraction_theta_max 0.871
_diffrn_measured_fraction_theta_full 1.000
_diffrn_reflns_Laue_measured_fraction_max 0.871
_diffrn_reflns_Laue_measured_fraction_full 1.000
_diffrn_reflns_point_group_measured_fraction_max 0.871
_diffrn_reflns_point_group_measured_fraction_full 1.000
_reflns_number_total 4996
_reflns_number_gt 3212
_reflns_threshold_expression 'I > 2\s(I)'
_reflns_Friedel_coverage 0.000
_reflns_Friedel_fraction_max .
_reflns_Friedel_fraction_full .

_reflns_special_details
;
_reflns_Friedel_fraction is defined as the number of unique
Friedel pairs measured divided by the number that would be
possible theoretically, ignoring centric projections and
systematic absences.
;

_computing_data_collection
;
CrysAlisPro, Agilent Technologies,
Version 1.171.37.35h (release 09-02-2015 CrysAlis171 .NET)
(compiled Feb 9 2015,16:26:32)
;
_computing_cell_refinement
;
CrysAlisPro, Agilent Technologies,
Version 1.171.37.35h (release 09-02-2015 CrysAlis171 .NET)
(compiled Feb 9 2015,16:26:32)
;
_computing_data_reduction
;
CrysAlisPro, Agilent Technologies,
Version 1.171.37.35h (release 09-02-2015 CrysAlis171 .NET)
(compiled Feb 9 2015,16:26:32)
;

_computing_structure_solution   'SHELXS-2013/1 (Sheldrick, 2013)'
_computing_structure_refinement   'SHELXL-2014/7 (Sheldrick, 2014)'
_computing_molecular_graphics   'ORTEP3 for Windows (Farrugia, 2012)'
_computing_publication_material
;
SHELXL-2014/7 (Sheldrick, 2014) and WINGX (Farrugia, 2012)
;
_refine_special_details
;
 Refined as a 2-component twin.
;
_refine_ls_structure_factor_coef Fsqd
_refine_ls_matrix_type   full
_refine_ls_weighting_scheme   calc
_refine_ls_weighting_details
'w=1/[\s^2^(Fo^2^)+(0.0582P)^2^] where P=(Fo^2^+2Fc^2^)/3'
_atom_sites_solution_primary   refxyz
_atom_sites_solution_secondary   ?
_atom_sites_solution_hydrogens   mixed
_refine_ls_hydrogen_treatment   mixed
_refine_ls_extinction_method   'SHELXL-2014/7 (Sheldrick 2014'
_refine_ls_extinction_coef   0.019(4)
_refine_ls_extinction_expression
 'Fc^*^=kFc[1+0.001xFc^2^\l^3^/sin(2\q)]^-1/4^'
_refine_ls_number_reflns   4996
_refine_ls_number_parameters   195
_refine_ls_number_restraints   0
_refine_ls_R_factor_all   0.0666
_refine_ls_R_factor_gt   0.0397
_refine_ls_wR_factor_ref   0.1079
_refine_ls_wR_factor_gt   0.1025
_refine_ls_goodness_of_fit_ref   0.903
_refine_ls_restrained_S_all   0.903
_refine_ls_shift/su_max   0.000
_refine_ls_shift/su_mean   0.000

loop_
 _atom_site_label
 _atom_site_type_symbol
 _atom_site_fract_x
 _atom_site_fract_y
 _atom_site_fract_z
 _atom_site_U_iso_or_equiv
 _atom_site_adp_type
 _atom_site_occupancy
 _atom_site_site_symmetry_order
 _atom_site_calc_flag
 _atom_site_refinement_flags_posn
 _atom_site_refinement_flags_adp
 _atom_site_refinement_flags_occupancy
 _atom_site_disorder_assembly

_atom_site_disorder_group
Cl42 Cl 0.00899(11) 0.27775(10) 0.11464(7) 0.0529(3) Uani 1 1 d . . . . .
Cl44 Cl 0.26017(13) -0.21174(9) 0.47456(8) 0.0683(3) Uani 1 1 d . . . . .
S5 S -0.29281(10) 0.59255(11) 0.30994(8) 0.0534(3) Uani 1 1 d . . . . .
N1 N -0.0820(3) 0.7488(3) 0.2145(2) 0.0410(6) Uani 1 1 d . . . . .
H1 H -0.177(4) 0.858(4) 0.193(3) 0.062 Uiso 1 1 d . U . . .
N2 N 0.0913(3) 0.7240(3) 0.1962(2) 0.0429(6) Uani 1 1 d . . . . .
N4 N 0.0604(3) 0.4946(2) 0.27551(19) 0.0314(6) Uani 1 1 d . . . . .
N63 N 0.6761(3) 0.0727(3) 0.1457(3) 0.0576(8) Uani 1 1 d . . . . .
C3 C 0.1758(4) 0.5688(3) 0.2346(3) 0.0349(7) Uani 1 1 d . . . . .
C5 C -0.1071(4) 0.6134(3) 0.2644(2) 0.0362(7) Uani 1 1 d . . . . .
C31 C 0.3676(4) 0.4853(3) 0.2409(3) 0.0439(8) Uani 1 1 d . . . . .
H311 H 0.3943 0.4422 0.3240 0.066 Uiso 1 1 calc R U . . .
H312 H 0.4169 0.5676 0.2165 0.066 Uiso 1 1 calc R U . . .
C41 C 0.1081(4) 0.3230(3) 0.3227(2) 0.0334(7) Uani 1 1 d . . . . .
C42 C 0.0891(4) 0.2125(3) 0.2562(3) 0.0360(7) Uani 1 1 d . . . . .
C43 C 0.1371(4) 0.0472(3) 0.3023(3) 0.0441(8) Uani 1 1 d . . . . .
H43 H 0.1234 -0.0274 0.2585 0.066 Uiso 1 1 calc R U . . .
C44 C 0.2054(4) -0.0054(3) 0.4136(3) 0.0465(8) Uani 1 1 d . . . . .
C45 C 0.2273(4) 0.1025(4) 0.4804(3) 0.0543(9) Uani 1 1 d . . . . .
H45 H 0.2750 0.0650 0.5553 0.081 Uiso 1 1 calc R U . . .
C46 C 0.1769(4) 0.2677(4) 0.4336(3) 0.0448(8) Uani 1 1 d . . . . .
H46 H 0.1897 0.3422 0.4779 0.067 Uiso 1 1 calc R U . . .
C61 C 0.4547(3) 0.3447(3) 0.1641(3) 0.0353(7) Uani 1 1 d . . . . .
C62 C 0.5869(4) 0.2000(4) 0.2084(3) 0.0486(8) Uani 1 1 d . . . . .
H62 H 0.6154 0.1911 0.2874 0.073 Uiso 1 1 calc R U . . .
C64 C 0.6335(5) 0.0882(4) 0.0334(4) 0.0595(10) Uani 1 1 d . . . . .
H64 H 0.6953 0.0008 -0.0121 0.089 Uiso 1 1 calc R U . . .
C65 C 0.5033(5) 0.2257(4) -0.0198(3) 0.0570(10) Uani 1 1 d . . . . .
H65 H 0.4767 0.2312 -0.0988 0.085 Uiso 1 1 calc R U . . .
C66 C 0.4135(4) 0.3551(4) 0.0480(3) 0.0475(8) Uani 1 1 d . . . . .
H66 H 0.3245 0.4500 0.0147 0.071 Uiso 1 1 calc R U . . .

loop_
_atom_site_aniso_label
_atom_site_aniso_U_11
_atom_site_aniso_U_22
_atom_site_aniso_U_33
_atom_site_aniso_U_23
_atom_site_aniso_U_13
_atom_site_aniso_U_12
Cl42 0.0638(6) 0.0486(5) 0.0469(5) -0.0087(4) -0.0159(4) -0.0184(4)
Cl44 0.0888(8) 0.0345(5) 0.0711(6) 0.0088(4) -0.0027(5) -0.0192(5)
S5 0.0338(5) 0.0647(6) 0.0644(6) -0.0103(5) -0.0028(4) -0.0211(4)
N1 0.0307(15) 0.0252(14) 0.0610(18) -0.0079(12) -0.0058(13) -0.0029(12)
N2 0.0355(15) 0.0261(14) 0.0663(18) -0.0085(12) -0.0017(13) -0.0106(12)
N4 0.0280(13) 0.0239(12) 0.0431(14) -0.0052(10) -0.0061(11) -0.0097(11)
N63 0.0444(18) 0.0308(15) 0.083(2) -0.0055(15) -0.0057(16) -0.0003(13)
C3 0.0313(17) 0.0270(16) 0.0488(19) -0.0097(13) -0.0042(14) -0.0117(14)
C5 0.0324(17) 0.0324(16) 0.0438(18) -0.0109(13) -0.0037(14) -0.0101(14)
C31 0.0327(18) 0.0309(16) 0.070(2) -0.0099(15) -0.0072(16) -0.0119(14)

C41 0.0351(17) 0.0288(15) 0.0390(17) -0.0057(13) -0.0021(13) -0.0147(13)
C42 0.0349(18) 0.0330(16) 0.0404(18) -0.0068(14) -0.0028(14) -0.0128(14)
C43 0.051(2) 0.0370(18) 0.049(2) -0.0127(15) 0.0029(16) -0.0219(16)
C44 0.053(2) 0.0297(16) 0.051(2) 0.0025(15) -0.0003(16) -0.0131(15)
C45 0.067(2) 0.047(2) 0.042(2) 0.0024(16) -0.0130(17) -0.0157(18)
C46 0.054(2) 0.0385(17) 0.045(2) -0.0080(15) -0.0109(16) -0.0183(16)
C61 0.0241(16) 0.0267(16) 0.053(2) -0.0001(14) -0.0023(14) -0.0098(13)
C62 0.0398(19) 0.0339(18) 0.061(2) -0.0014(16) -0.0067(16) -0.0039(15)
C64 0.061(3) 0.046(2) 0.072(3) -0.0208(19) 0.011(2) -0.0204(19)
C65 0.066(3) 0.049(2) 0.058(2) -0.0047(19) -0.002(2) -0.026(2)
C66 0.043(2) 0.0310(17) 0.064(2) 0.0071(16) -0.0125(17) -0.0121(15)

_geom_special_details
;
 All esds (except the esd in the dihedral angle between two l.s. planes)
 are estimated using the full covariance matrix. The cell esds are taken
 into account individually in the estimation of esds in distances, angles
 and torsion angles; correlations between esds in cell parameters are only
 used when they are defined by crystal symmetry. An approximate (isotropic)
 treatment of cell esds is used for estimating esds involving l.s. planes.
;

loop_
 _geom_bond_atom_site_label_1
 _geom_bond_atom_site_label_2
 _geom_bond_distance
 _geom_bond_site_symmetry_2
 _geom_bond_publ_flag
Cl42 C42 1.725(3) . ?
Cl44 C44 1.742(3) . ?
S5 C5 1.665(3) . ?
N1 C5 1.332(3) . ?
N1 N2 1.381(3) . ?
N1 H1 0.99(3) . ?
N2 C3 1.296(3) . ?
N4 C5 1.381(3) . ?
N4 C3 1.384(3) . ?
N4 C41 1.436(3) . ?
N63 C64 1.325(4) . ?
N63 C62 1.329(4) . ?
C3 C31 1.484(4) . ?
C31 C61 1.508(4) . ?
C31 H311 0.9700 . ?
C31 H312 0.9700 . ?
C41 C46 1.372(4) . ?
C41 C42 1.385(3) . ?
C42 C43 1.384(4) . ?
C43 C44 1.372(4) . ?
C43 H43 0.9300 . ?
C44 C45 1.380(4) . ?
C45 C46 1.383(4) . ?

C45 H45 0.9300 . ?
C46 H46 0.9300 . ?
C61 C66 1.370(4) . ?
C61 C62 1.380(4) . ?
C62 H62 0.9300 . ?
C64 C65 1.374(5) . ?
C64 H64 0.9300 . ?
C65 C66 1.377(5) . ?
C65 H65 0.9300 . ?
C66 H66 0.9300 . ?

loop_
 _geom_angle_atom_site_label_1
 _geom_angle_atom_site_label_2
 _geom_angle_atom_site_label_3
 _geom_angle
 _geom_angle_site_symmetry_1
 _geom_angle_site_symmetry_3
 _geom_angle_publ_flag
C5 N1 N2 113.6(2) . . ?
C5 N1 H1 124.0(17) . . ?
N2 N1 H1 122.4(17) . . ?
C3 N2 N1 104.8(2) . . ?
C5 N4 C3 108.5(2) . . ?
C5 N4 C41 126.1(2) . . ?
C3 N4 C41 125.4(2) . . ?
C64 N63 C62 116.8(3) . . ?
N2 C3 N4 110.1(2) . . ?
N2 C3 C31 125.0(2) . . ?
N4 C3 C31 124.8(2) . . ?
N1 C5 N4 103.0(2) . . ?
N1 C5 S5 129.1(2) . . ?
N4 C5 S5 127.8(2) . . ?
C3 C31 C61 114.8(2) . . ?
C3 C31 H311 108.6 . . ?
C61 C31 H311 108.6 . . ?
C3 C31 H312 108.6 . . ?
C61 C31 H312 108.6 . . ?
H311 C31 H312 107.5 . . ?
C46 C41 C42 119.8(2) . . ?
C46 C41 N4 119.5(2) . . ?
C42 C41 N4 120.7(2) . . ?
C43 C42 C41 120.1(3) . . ?
C43 C42 Cl42 119.3(2) . . ?
C41 C42 Cl42 120.7(2) . . ?
C44 C43 C42 119.1(3) . . ?
C44 C43 H43 120.4 . . ?
C42 C43 H43 120.4 . . ?
C43 C44 C45 121.6(3) . . ?
C43 C44 Cl44 119.5(2) . . ?
C45 C44 Cl44 118.8(3) . . ?

C44 C45 C46 118.6(3) . . ?
C44 C45 H45 120.7 . . ?
C46 C45 H45 120.7 . . ?
C41 C46 C45 120.8(3) . . ?
C41 C46 H46 119.6 . . ?
C45 C46 H46 119.6 . . ?
C66 C61 C62 117.0(3) . . ?
C66 C61 C31 122.9(3) . . ?
C62 C61 C31 120.0(3) . . ?
N63 C62 C61 124.3(3) . . ?
N63 C62 H62 117.8 . . ?
C61 C62 H62 117.8 . . ?
N63 C64 C65 123.9(4) . . ?
N63 C64 H64 118.0 . . ?
C65 C64 H64 118.0 . . ?
C64 C65 C66 117.6(4) . . ?
C64 C65 H65 121.2 . . ?
C66 C65 H65 121.2 . . ?
C61 C66 C65 120.3(3) . . ?
C61 C66 H66 119.8 . . ?
C65 C66 H66 119.8 . . ?

loop_
 _geom_torsion_atom_site_label_1
 _geom_torsion_atom_site_label_2
 _geom_torsion_atom_site_label_3
 _geom_torsion_atom_site_label_4
 _geom_torsion
 _geom_torsion_site_symmetry_1
 _geom_torsion_site_symmetry_2
 _geom_torsion_site_symmetry_3
 _geom_torsion_site_symmetry_4
 _geom_torsion_publ_flag
C5 N1 N2 C3 0.7(3) . . . . ?
N1 N2 C3 N4 0.6(3) . . . . ?
N1 N2 C3 C31 -175.9(3) . . . . ?
C5 N4 C3 N2 -1.6(3) . . . . ?
C41 N4 C3 N2 179.0(2) . . . . ?
C5 N4 C3 C31 174.9(3) . . . . ?
C41 N4 C3 C31 -4.5(4) . . . . ?
N2 N1 C5 N4 -1.6(3) . . . . ?
N2 N1 C5 S5 175.3(2) . . . . ?
C3 N4 C5 N1 1.9(3) . . . . ?
C41 N4 C5 N1 -178.7(2) . . . . ?
C3 N4 C5 S5 -175.0(2) . . . . ?
C41 N4 C5 S5 4.4(4) . . . . ?
N2 C3 C31 C61 -118.1(3) . . . . ?
N4 C3 C31 C61 65.9(4) . . . . ?
C5 N4 C41 C46 -108.0(3) . . . . ?
C3 N4 C41 C46 71.3(4) . . . . ?
C5 N4 C41 C42 73.5(4) . . . . ?

C3 N4 C41 C42 -107.2(3) . . . . ?
C46 C41 C42 C43 0.8(4) . . . . ?
N4 C41 C42 C43 179.4(2) . . . . ?
C46 C41 C42 Cl42 -178.1(2) . . . . ?
N4 C41 C42 Cl42 0.5(4) . . . . ?
C41 C42 C43 C44 -0.7(4) . . . . ?
Cl42 C42 C43 C44 178.2(2) . . . . ?
C42 C43 C44 C45 -0.1(5) . . . . ?
C42 C43 C44 Cl44 178.0(2) . . . . ?
C43 C44 C45 C46 0.8(5) . . . . ?
Cl44 C44 C45 C46 -177.4(2) . . . . ?
C42 C41 C46 C45 -0.1(5) . . . . ?
N4 C41 C46 C45 -178.7(3) . . . . ?
C44 C45 C46 C41 -0.7(5) . . . . ?
C3 C31 C61 C66 44.0(4) . . . . ?
C3 C31 C61 C62 -138.6(3) . . . . ?
C64 N63 C62 C61 0.1(5) . . . . ?
C66 C61 C62 N63 0.5(5) . . . . ?
C31 C61 C62 N63 -177.0(3) . . . . ?
C62 N63 C64 C65 -0.7(5) . . . . ?
N63 C64 C65 C66 0.6(6) . . . . ?
C62 C61 C66 C65 -0.6(4) . . . . ?
C31 C61 C66 C65 176.8(3) . . . . ?
C64 C65 C66 C61 0.1(5) . . . . ?

_refine_diff_density_max   0.263
_refine_diff_density_min   -0.250
_refine_diff_density_rms   0.072

_shelx_res_file
;

    shelx.res created by SHELXL-2014/7

TITL T17 in P-1
CELL 0.71073   8.4234   8.8584 11.2513   81.356   83.534   66.032
ZERR   2.00   0.0010   0.0009   0.0020   0.013   0.012   0.011
LATT   1
SFAC C   H   N   S   CL
UNIT 28 20 8 2 4
MERG   0
FMAP   2
ACTA
BOND $H
CONF
L.S. 40
PLAN -2
WGHT   0.058200
EXTI   0.018621
BASF   0.43793

```
FVAR       5.87660
CL42  5    0.008987    0.277750    0.114641    11.00000    0.06378    0.04856 =
        0.04694   -0.00870   -0.01592   -0.01844
CL44  5    0.260172   -0.211735    0.474562    11.00000    0.08883    0.03452 =
        0.07105    0.00877   -0.00268   -0.01920
S5    4   -0.292806    0.592551    0.309938    11.00000    0.03376    0.06467 =
        0.06436   -0.01031   -0.00279   -0.02114
N1    3   -0.081959    0.748820    0.214471    11.00000    0.03070    0.02521 =
        0.06097   -0.00794   -0.00583   -0.00290
H1    2   -0.177457    0.858046    0.192663    11.00000   -1.50000
N2    3    0.091303    0.724038    0.196216    11.00000    0.03547    0.02612 =
        0.06631   -0.00853   -0.00173   -0.01063
N4    3    0.060391    0.494564    0.275511    11.00000    0.02801    0.02387 =
        0.04315   -0.00519   -0.00611   -0.00974
N63   3    0.676106    0.072723    0.145731    11.00000    0.04444    0.03081 =
        0.08333   -0.00553   -0.00574   -0.00034

C3    1    0.175794    0.568754    0.234591    11.00000    0.03129    0.02702 =
        0.04883   -0.00972   -0.00419   -0.01168
C5    1   -0.107077    0.613449    0.264356    11.00000    0.03242    0.03237 =
        0.04381   -0.01091   -0.00373   -0.01014
C31   1    0.367567    0.485272    0.240914    11.00000    0.03272    0.03089 =
        0.06995   -0.00993   -0.00717   -0.01192
AFIX  23
H311  2    0.394293    0.442206    0.324017    11.00000   -1.50000
H312  2    0.416930    0.567624    0.216549    11.00000   -1.50000
AFIX   0
C41   1    0.108110    0.322973    0.322657    11.00000    0.03510    0.02879 =
        0.03901   -0.00571   -0.00211   -0.01468
C42   1    0.089052    0.212459    0.256222    11.00000    0.03493    0.03303 =
        0.04039   -0.00680   -0.00280   -0.01281
C43   1    0.137148    0.047155    0.302315    11.00000    0.05148    0.03697 =
        0.04935   -0.01269    0.00288   -0.02187
AFIX  43
H43   2    0.123435   -0.027359    0.258482    11.00000   -1.50000
AFIX   0
C44   1    0.205359   -0.005386    0.413579    11.00000    0.05303    0.02966 =
        0.05064    0.00252   -0.00028   -0.01312
C45   1    0.227310    0.102517    0.480386    11.00000    0.06702    0.04690 =
        0.04213    0.00245   -0.01296   -0.01573
AFIX  43
H45   2    0.274991    0.064972    0.555250    11.00000   -1.50000
AFIX   0
C46   1    0.176887    0.267666    0.433626    11.00000    0.05409    0.03855 =
        0.04473   -0.00801   -0.01087   -0.01826
AFIX  43
H46   2    0.189740    0.342179    0.477922    11.00000   -1.50000
AFIX   0
C61   1    0.454652    0.344724    0.164084    11.00000    0.02405    0.02670 =
        0.05332   -0.00005   -0.00230   -0.00980
C62   1    0.586891    0.200032    0.208447    11.00000    0.03981    0.03386 =
```

```
        0.06124   -0.00142   -0.00672   -0.00387
AFIX  43
H62   2    0.615414    0.191068    0.287419    11.00000   -1.50000
AFIX   0
C64   1    0.633451    0.088206    0.033415    11.00000    0.06068    0.04568 =
        0.07234   -0.02078    0.01103   -0.02037
AFIX  43
H64   2    0.695308    0.000801   -0.012128    11.00000   -1.50000
AFIX   0
C65   1    0.503254    0.225714   -0.019816    11.00000    0.06577    0.04936 =
        0.05778   -0.00474   -0.00235   -0.02559
AFIX  43
H65   2    0.476729    0.231186   -0.098796    11.00000   -1.50000
AFIX   0
C66   1    0.413493    0.355082    0.047964    11.00000    0.04295    0.03096 =
        0.06434    0.00706   -0.01250   -0.01212
AFIX  43
H66   2    0.324488    0.449954    0.014741    11.00000   -1.50000
AFIX   0
HKLF    5

REM  T17 in P-1
REM R1 =  0.0397 for    3212 Fo > 4sig(Fo)  and  0.0666 for all    4996 data
REM    195 parameters refined using      0 restraints

END

WGHT      0.0582      0.0000

REM Highest difference peak  0.263,  deepest hole -0.250,  1-sigma level  0.072
Q1    1  -0.4070  0.5775  0.2774  11.00000  0.05    0.26
Q2    1   0.5058  0.1623  0.0052  11.00000  0.05    0.23
;
```

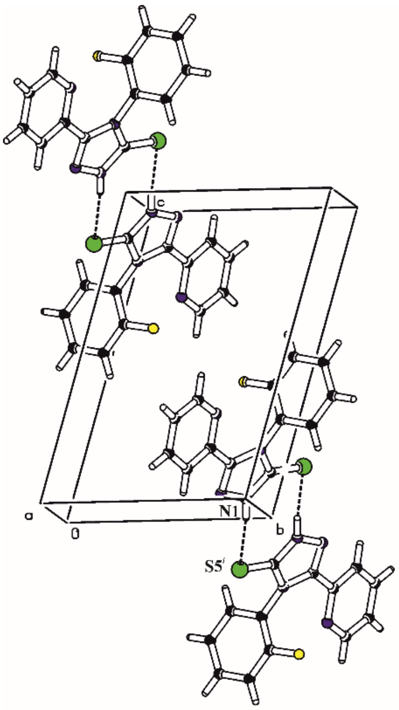

C1

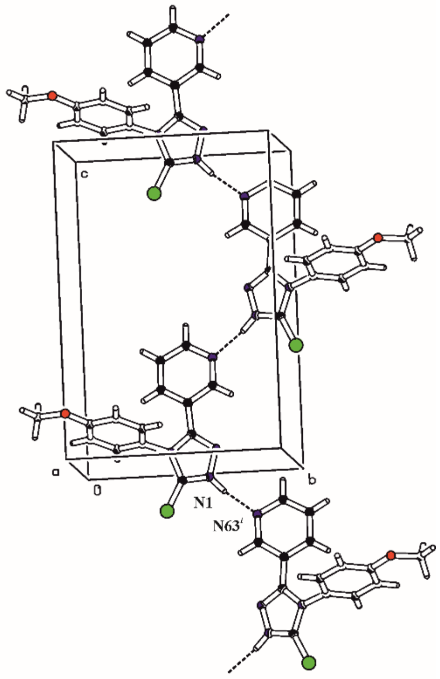

C12

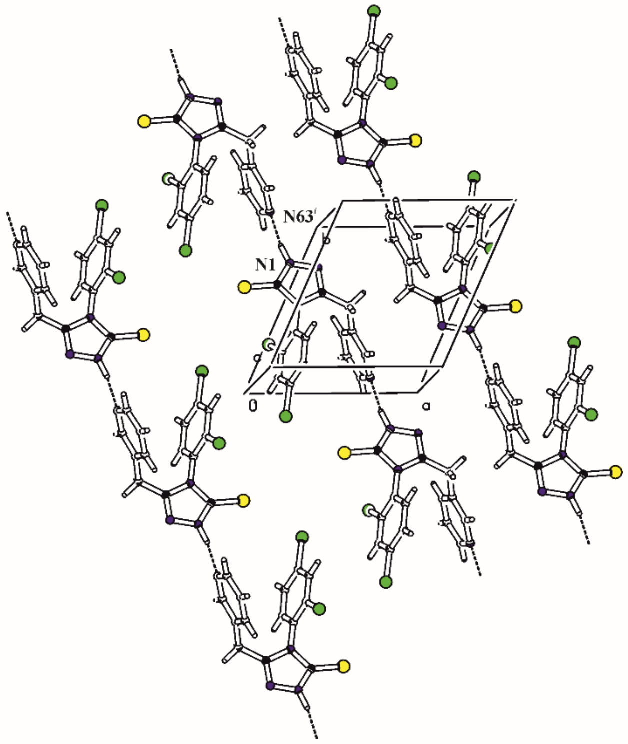

C13

Figure 1S. The molecular packing of **C1, C12** and **C13**. Dashed lines indicate intermolecular hydrogen bonds; **C1** *i* = -*x*, 2-*y*, -*z*; **C12:** *i* = *x*, 3/2-*y*, -1/2+*z*; **C13**: *i* = -1+*x*, 1+*y*, *z*.

MS analysis for new compounds

Table 1S.

LP.	Compound formula, thoretical and measured monoisotopic mass, and mass difference in ppm	Theoretical monoisotopic mass of protonated ion and acquired LC/QTOF HRMS spectra
B13	C14H12N4OSCl2<br>Calculated monoisotopic mass: 354.0109<br><br>Measured monoisotopic mass: 354.0122<br><br>Mass error: 3.84 ppm	Theoretical monoisotopic mass of $[M+H]^+$: 355.0182<br>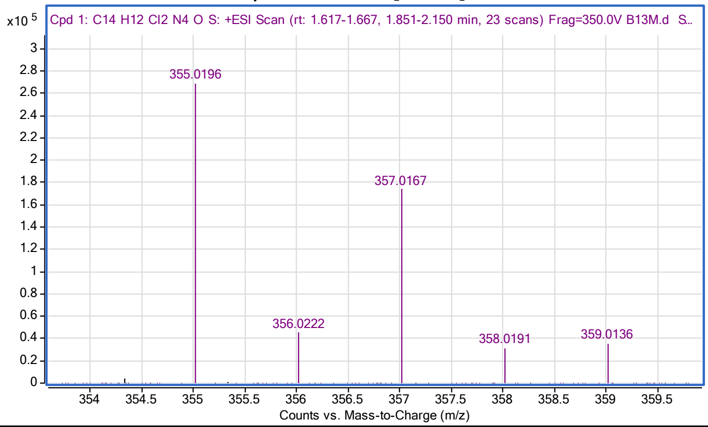
B14	C14H12ON4SCl2<br>Calculated monoisotopic mass: 354.0109<br><br>Measured monoisotopic mass: 354.012<br><br>Mass error: 3.13 ppm	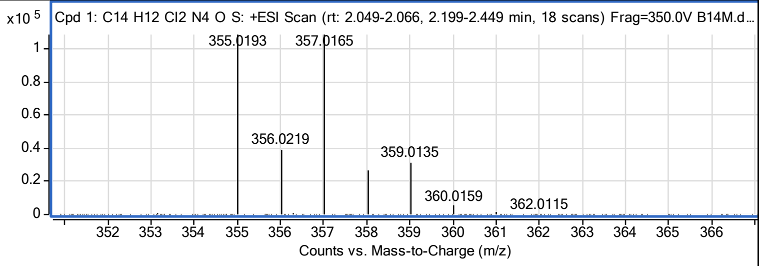
C13	C14H10N4SCl2<br>Calculated monoisotopic mass: 336.0003<br>Measured monoisotopic mass: 336.0017<br><br>Mass error: 3.96 ppm	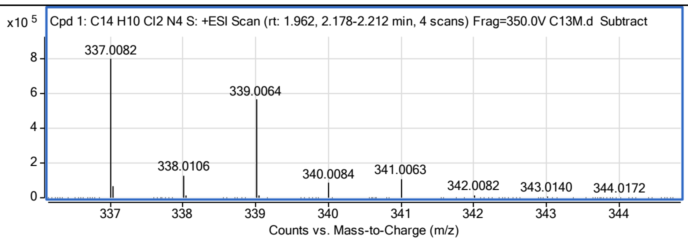
C14	C14H10N4SCl2<br>Calculated monoisotopic mass: 336.0003<br><br>Measured monoisotopic mass: 336.0006<br><br>Mass error: 0.78 ppm	Theoretical monoisotopic mass of $[M+H]^+$: 337.0076<br>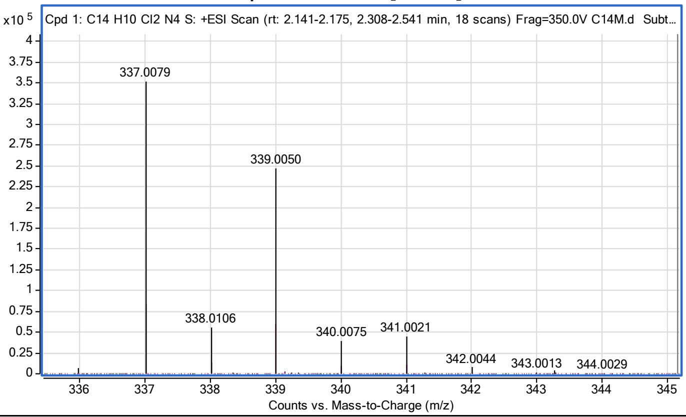

Theoretical calculation

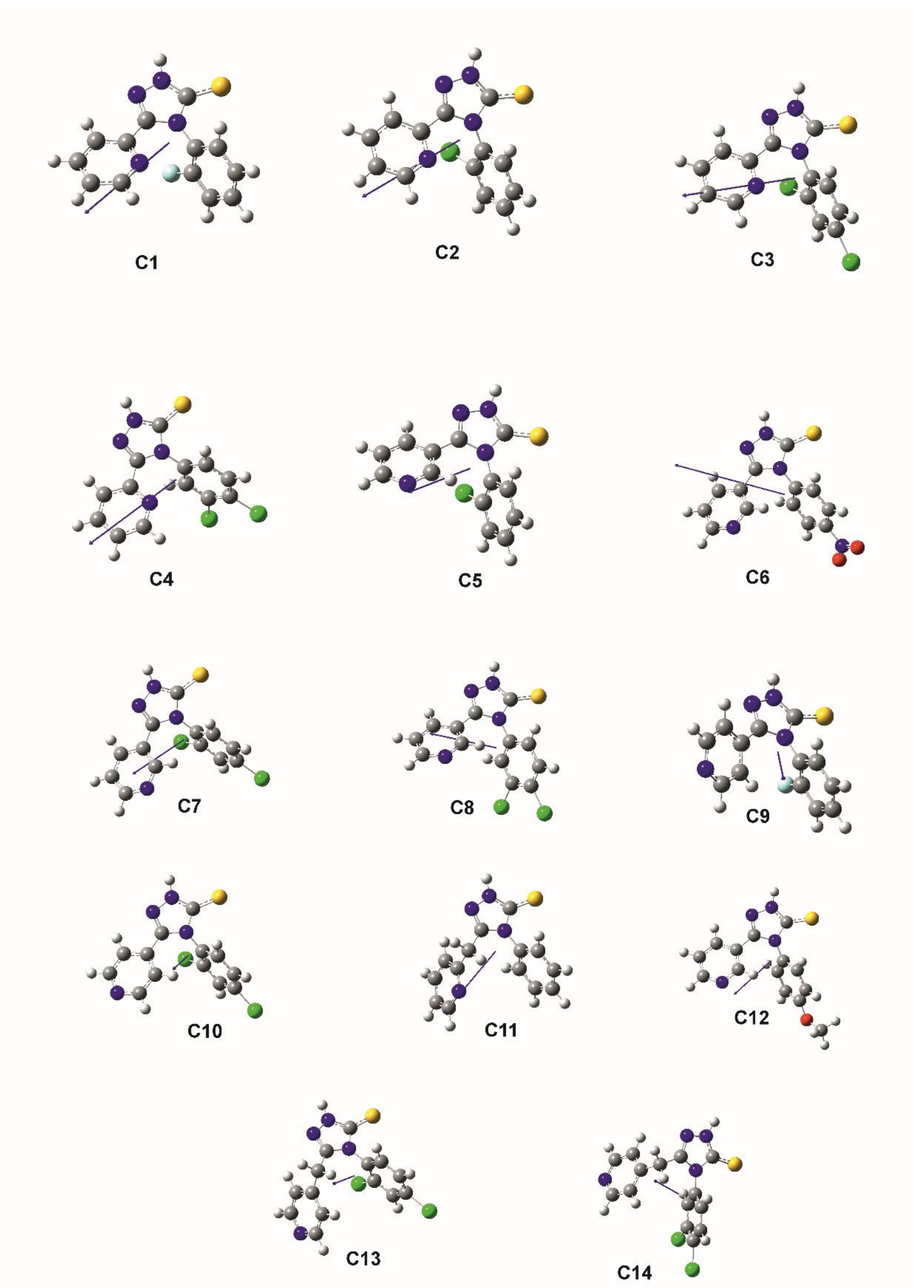

Figure 2S. A view of the molecules **C1**–**C14** with the vectors of dipole moments in the low-energy conformation calculated at the DFT/B3LYP/6-311++G(d,p) level.

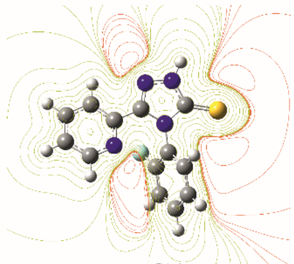

C1

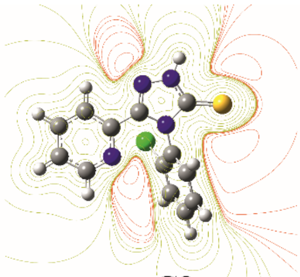

C2

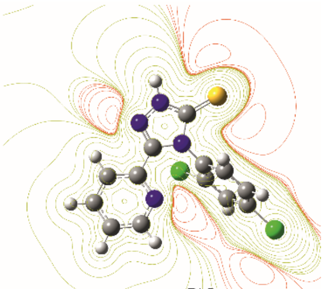

C3

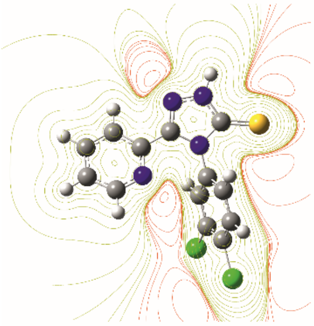

C4

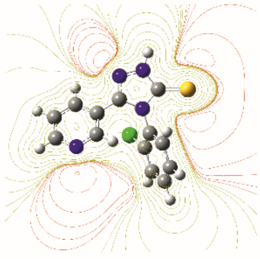

C5

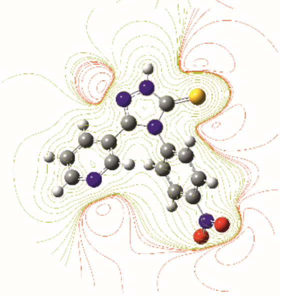

C6

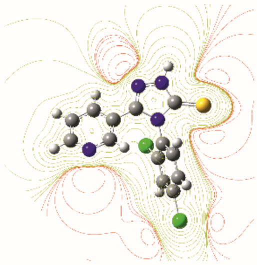

C7

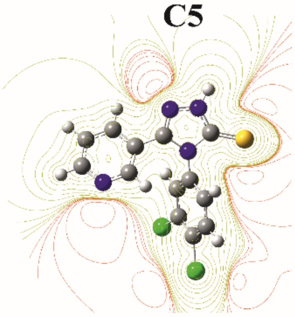

C8

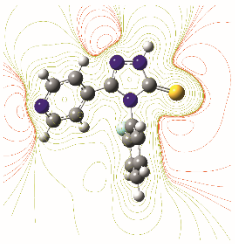

C9

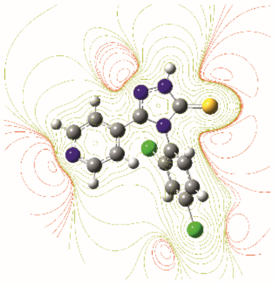

C10

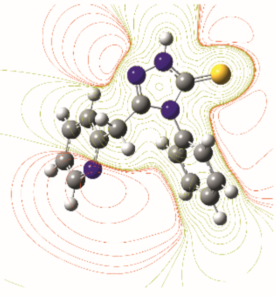

C11

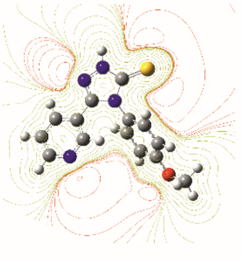

C12

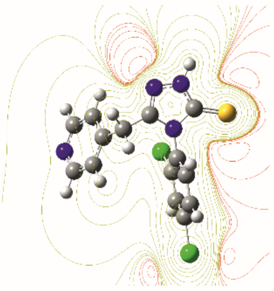

C13

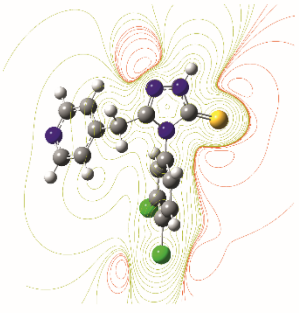

C14

Fig. 3S. The contour maps of molecular electrostatic potential for compounds **C1–C14** in the plane of 1,2,4-triazole ring; the red lines represent regions with the negative electrostatic potential and yellow lines represent regions with the positive electrostatic potential.

Molecular docking

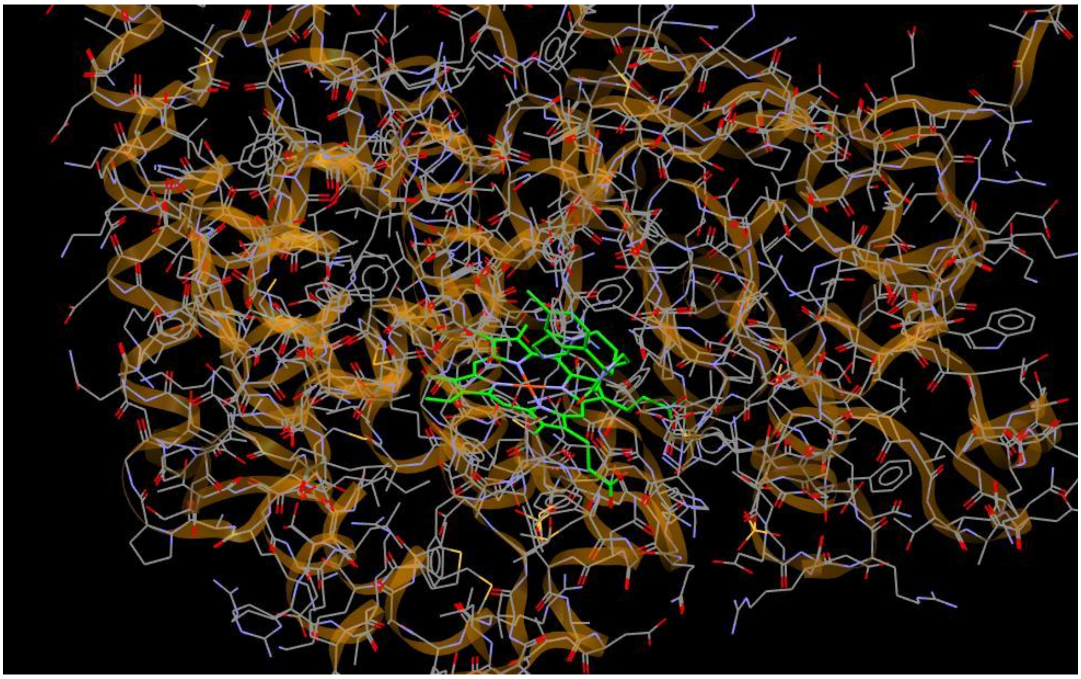

Fig. 4S. The crystal structure of the cytochrome P450 CYP121 of Mycobacterium tuberculosis obtained from expression system *Escherichia coli* in complex with 1-[(4-chlorophenyl)methyl]-4-(3-imidazol-1-ylpropyl)piperazin-2-one (9KE) and heme.

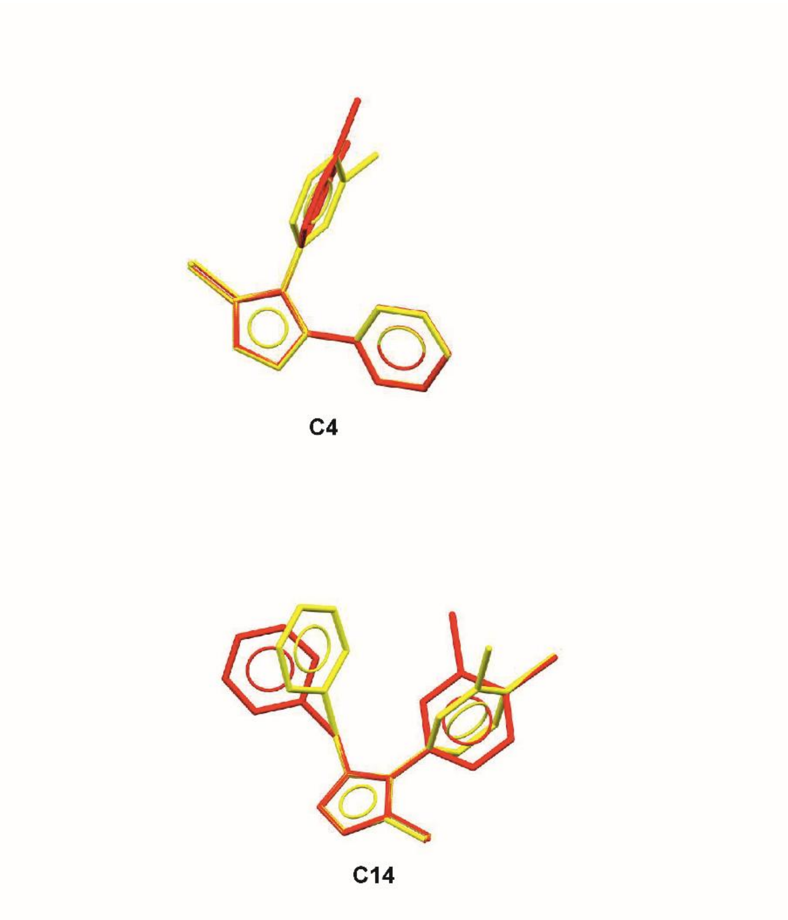

Fig. 5S. The overlay of molecules **C4** and **C14** in conformations observed in binding site of the cytochrome P450 CYP121 9red) and to their starting conformations for the docking process obtained as a result of energy minimization and energy optimization at DFT level (yellow).

Spectral data for new compounds:

Compound B13

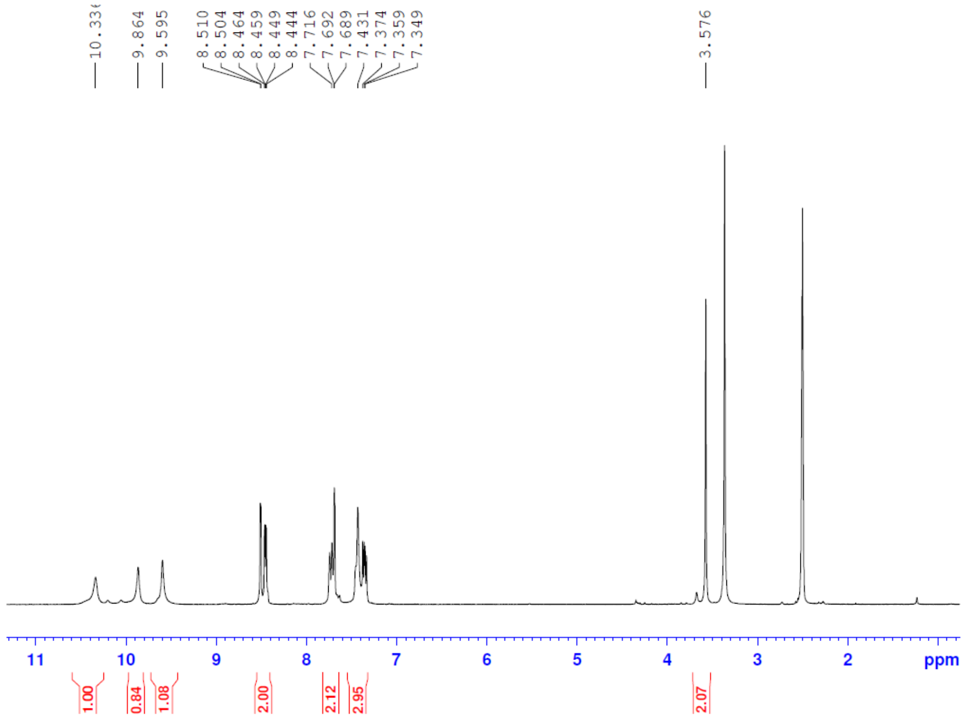

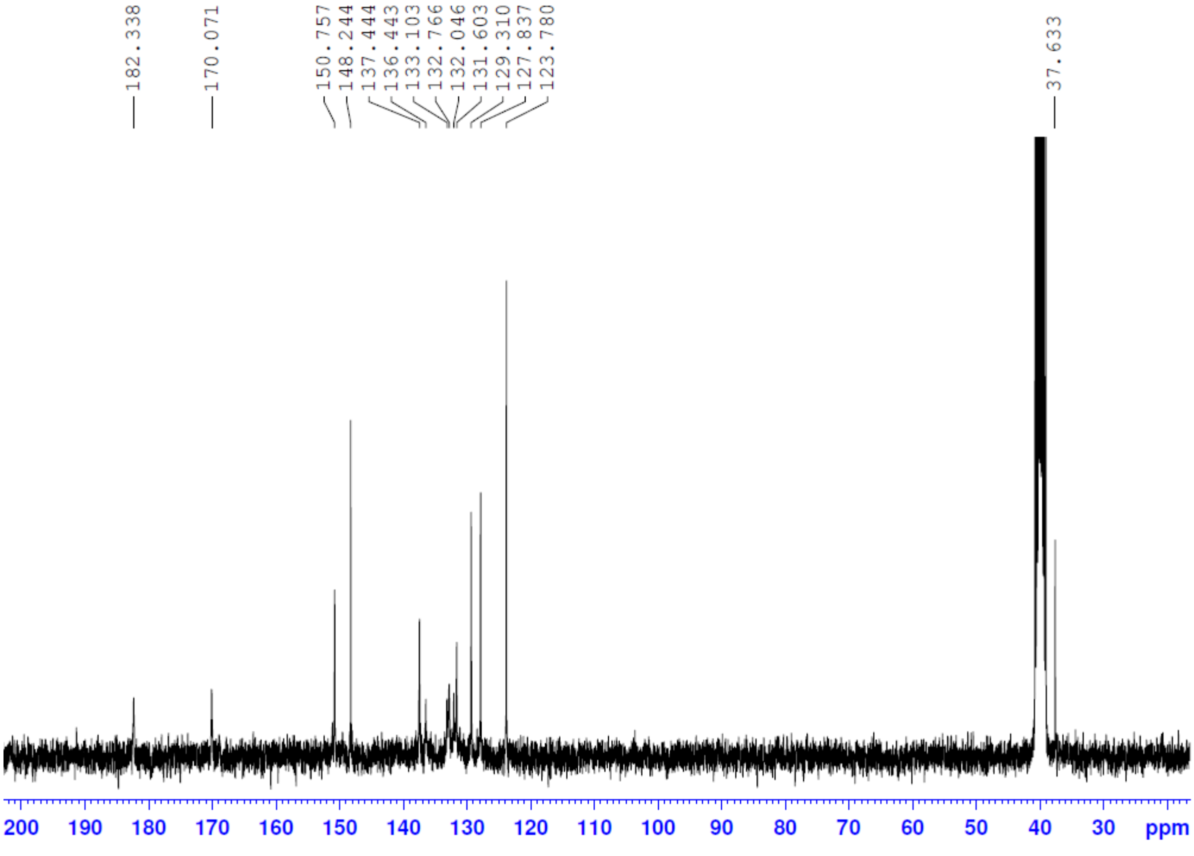

Compound B14

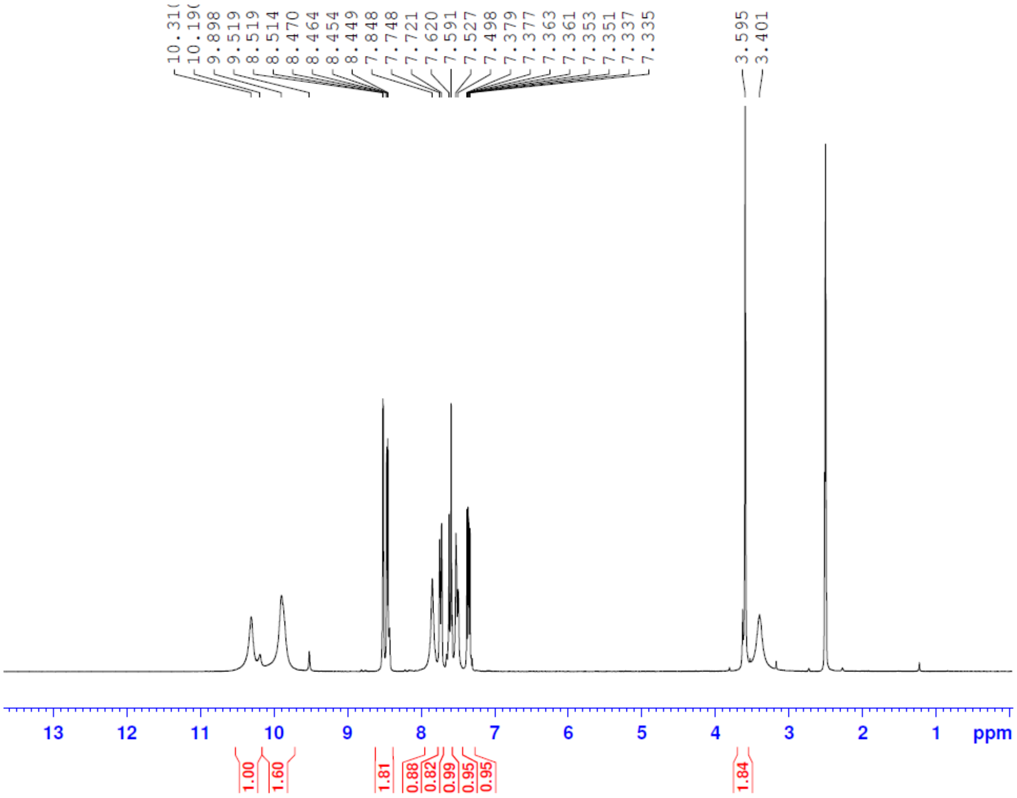

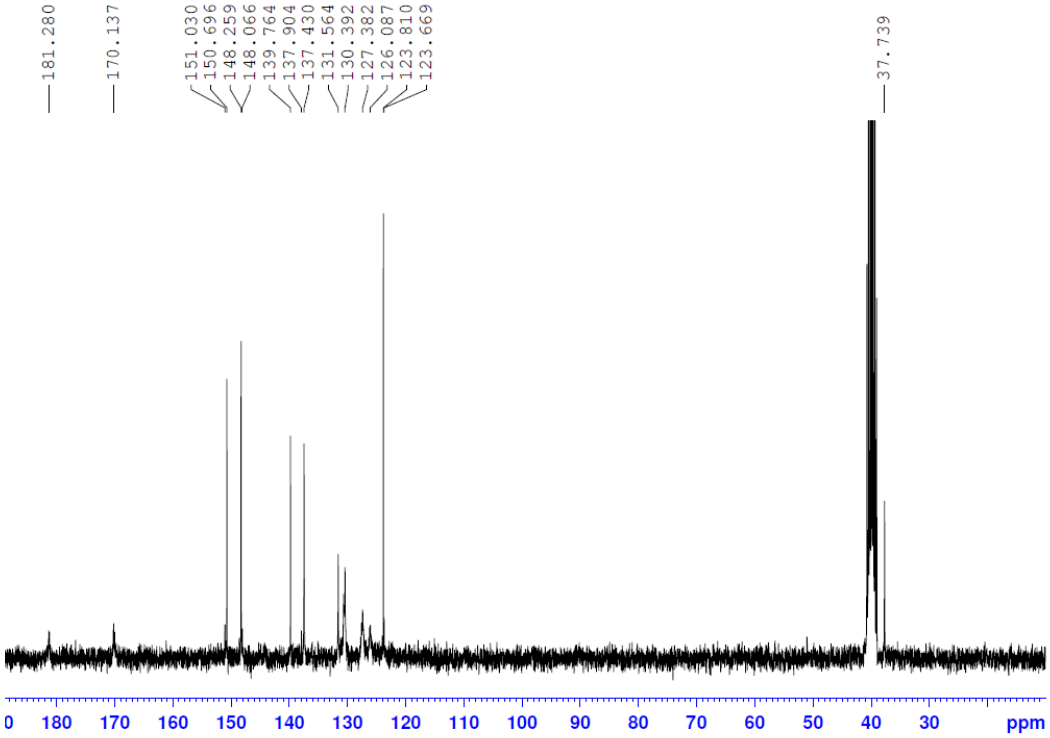

Compound C13

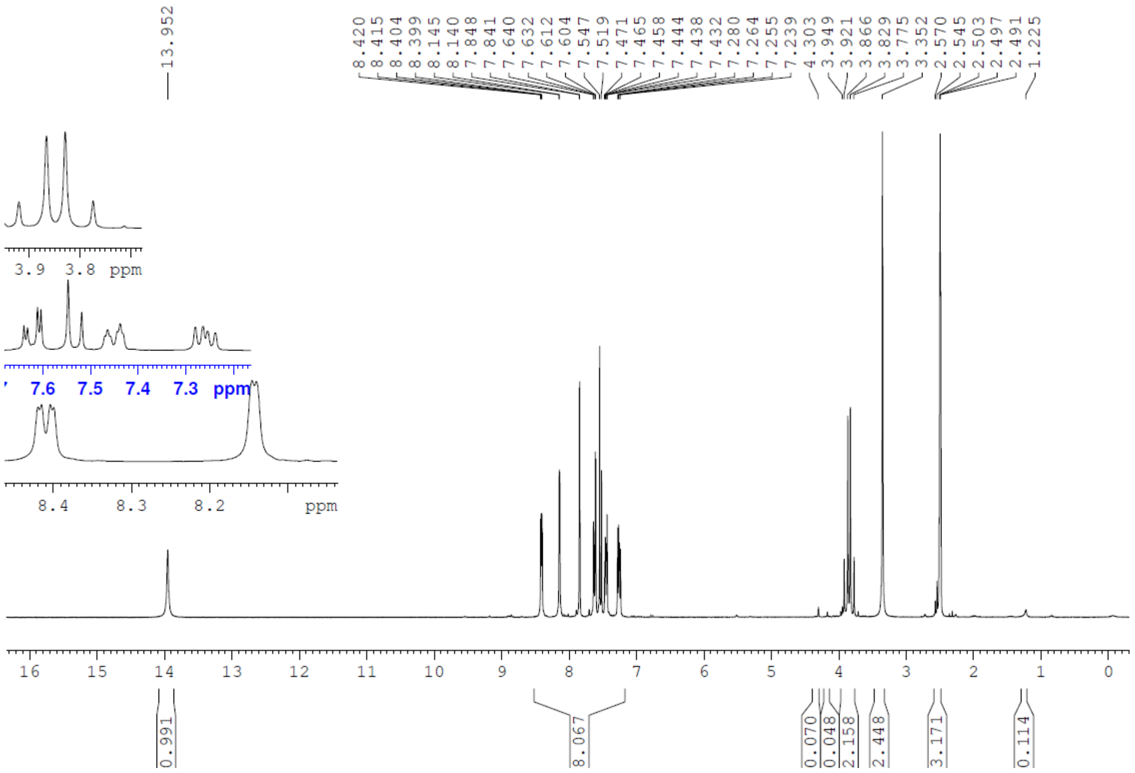

Compound C14

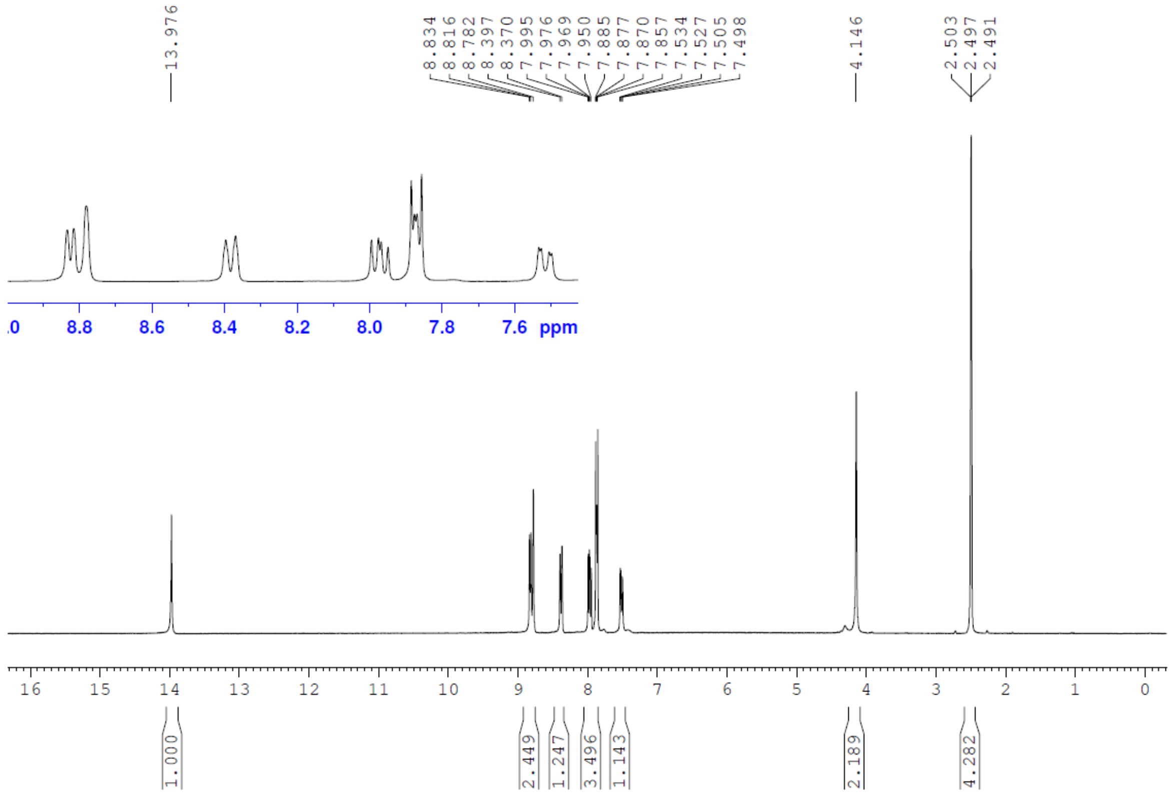

Supplement: Supplementary file 1 [file molecules-25-06033-s001.pdf]
